# Biological and glucocorticoids treatment impair the medium-term immunogenicity to SARS-CoV-2 mRNA vaccines in autoimmune inflammatory rheumatic diseases

**DOI:** 10.1186/s40001-023-01620-7

**Published:** 2024-01-05

**Authors:** Silvia Garcia-Cirera, Joan Calvet, Juan Francisco Delgado de la Poza, Antoni Berenguer-Llergo, Cristóbal Orellana, Menna Rusiñol, Maria Llop, Marta Arévalo, Alba Garcia-Pinilla, Ester Costa, Cristina Aymerich, Rafael Gómez, Anna Carreras, Jordi Gratacós

**Affiliations:** 1 Rheumatology Department, Parc Taulí Hospital Universitari. Institut d’Investigació I Innovació Parc Taulí (I3PT-CERCA), c/Parc Taulí S/N, Edifici VII Centenari, 08208 Sabadell, Spain; 2 Departament de Medicina, Universitat Autónoma de Barcelona (UAB), 08003 Barcelona, Spain; 3 Immunology Department, Parc Taulí Hospital Universitari. Institut d’Investigació I Innovació Parc Taulí (I3PT-CERCA), 08208 Sabadell, Spain; 4 Rheumatology Department, Biostatistics and Bioinformatics at Institut d’Investigació i Innovació Parc Taulí (I3PT-CERCA), 08028 Sabadell, Spain

**Keywords:** Autoimmune disease, Immune response, COVID19, Neutralizing antibodies

## Abstract

**Background:**

This study aims to assess the sustained immunological response to the SARS-CoV-2 vaccine in patients with autoimmune inflammatory rheumatic diseases (AIRD) undergoing different treatment regimens.

**Methods:**

We conducted a prospective observational study involving 157 AIRD patients without prior COVID-19 infection. Treatment regimens included non-treatment or glucocorticoid-only (not-treated/GCs), non-biological drugs, biological therapy, and JAK inhibitors. All participants completed the two-dose vaccine schedule, and 110 of them received an additional booster dose. Serum samples were collected approximately 3–6 months after the second and third vaccine doses to measure antibodies against the Spike protein (antiS-AB) and neutralizing antibodies (nAB) targeting six SARS-CoV-2 variants.

**Results:**

Following the third dose, all patients exhibited a significant increase in antiS-AB (FC = 15, *p* < 0.0001). Patients under biological therapy had lower titres compared to the non-biological (66% decrease, *p* = 0.038) and the not-treated/GCs group (62% decrease, *p* = 0.0132), with the latter persisting after the booster dose (86% decrease, *p* = 0.0027). GC use was associated with lower antiS-AB levels in the biological group (87% decrease, *p* = 0.0124), although not statistically significant after confounders adjustment. nABs showed the highest positivity rates for the wild-type strain before (50%) and after the booster dose (93%), while the Omicron variant exhibited the lowest rates (11% and 55%, respectively). All variants demonstrated similar positivity patterns and good concordance with antiS-AB (AUCs from 0.896 to 0.997).

**Conclusions:**

The SARS-CoV-2 vaccine booster strategy effectively elicited a sustained antibody immune response in AIRD patients. However, patients under biological therapies exhibited a reduced response to the booster dose, particularly when combined with GCs.

**Supplementary Information:**

The online version contains supplementary material available at 10.1186/s40001-023-01620-7.

## Introduction.

Patients with autoimmune inflammatory rheumatic diseases (AIRD) are at a higher risk of infections, including COVID19, compared to the general population [1–3]. This increased susceptibility can be attributed to the inflammatory burden and comorbidities associated with their condition, as well as to treatments used to manage AIRD, including glucocorticoids, immunosuppressive agents, and immunomodulatory therapies [4, 5]. However, the specific contributions of the disease and these treatments to this imbalance in infection susceptibility remains unclear [6].

The introduction of SARS-CoV-2 vaccination dramatically decreased the severity of the infection. Previous research has documented the safety and effectiveness of SARS-CoV-2 vaccines in patients with AIRD [7–9]. Nevertheless, concerns persist regarding the durability of vaccine-induced protection in this population. Several studies, including a systematic review, have investigated the seroconversion and vaccine response among AIRD patients [10, 11], and have observed a rate improvement of this response following vaccination. These studies have also indicated that the medications prescribed to AIRD patients, particularly glucocorticoids, mycophenolate–mofetil, and rituximab, may have a greater impact on the diminished vaccine response than the underlying autoimmune disorder itself [12–14]. Moreover, it is important to note that, patients suffering from AIRD presented an impairment in both, the humoral and cellular immune induction response compared to healthy controls [15]. In line with previous research, our group has recently published a study highlighting the detrimental effect of glucocorticoids on the immune response of individuals with systemic lupus erythematosus (SLE), regardless of their treatment regimen, in comparison to healthy individuals [16]. Other works have reported a significant decline in the humoral immune response among AIRD patients 6 months after receiving the second dose of the vaccine [17].

The administration of a third dose (booster) of the SARS-CoV-2 vaccine has generally shown to enhance immunity in most individuals. However, a subset of patients with compromised immune systems may not attain an optimal immune response even after receiving a booster, highlighting the need for further research to identify the most effective approach for this specific population [18–20]. A recent study on AIRD patients observed that older age, vasculitis, and the use of medications such as prednisone, mycophenolate–mofetil, and biologic therapies (belimumab, rituximab, and abatacept) were associated with lower levels of IgG and neutralizing antibodies in the short term (1 month) after vaccination [18]. Another study found that rituximab and glucocorticoids were linked to a diminished humoral response, with no impact in the cellular immunity, to the third dose of the SARS-CoV-2 vaccine, consistent with previous research highlighting the negative impact of rituximab on immune protection in AIRD patients [19, 20]. Nevertheless, individuals with AIRD who received three vaccine doses still experienced less severe SARS-CoV-2 infections and a reduced risk of hospital admission compared to those who received two doses or remained unvaccinated [21].

Despite previous studies, there is still limited understanding of the medium-term response (3 and 6 months) to the SARS-CoV-2 vaccine in patients with AIRD, particularly in relation to the third dose and its correlation with the level of protection achieved after the second dose. The lack of comprehensive data hampers the development of strategies to ensure sustained protection against COVID-19 in this vulnerable population, and it remains unclear whether additional doses or alternative strategies are necessary to achieve long-term immunity.

Our study aims to address these limitations by evaluating the sustained response to the SARS-CoV-2 vaccine in patients with various AIRD who receive different therapy regimens. For this purpose, we assessed the levels of Spike (S) protein antibodies (antiS-AB) and neutralizing antibodies (nAB) against multiple SARS-CoV-2 variants between 3 and 6 months after the second and third vaccine doses. These antibody levels serve as a measure of protection against SARS-CoV-2 infection, allowing us to assess the medium-term effectiveness of the vaccine, identify individuals with impaired antibody immune response, and explore the potential benefits of the booster strategy in AIRD patients.

## Methods

### Study design and subjects

The present work is a prospective observational study aimed at comparing the immune response to the SARS-CoV-2 vaccine following the initial two-dose regimen and the booster (third dose) of BNT162b2 (Pfizer) or mRNA-1237 (Moderna) vaccines. We included 157 patients from the outpatient Rheumatology department diagnosed with Rheumatoid Arthritis (RA), Systemic Lupus Erythematosus (SLE), Giant Cell Arteritis (GCA), Psoriatic Arthritis (PsA), and Axial Spondylitis B27 + (SpA). These patients were on different treatment regimens, including no treatment or treatment with glucocorticoids only (not-treated/GCs), non-biological drugs (non-biological), biological therapy (biological), and JAK inhibitors (JAKi). Among the enrolled patients, 43 were using glucocorticoids (GCs) at the time of recruitment. Of them, 25 were on prednisone 5 mg/day or less, corresponding to patients with SLE [7], RA [11], and PsA [7]. The remaining 18 subjects were patients with GCA, of which only 6 patients were prescribed with more than 5 mg/day. None of the subjects had been previously exposed to SARS-CoV-2 infection before the two sample extractions. All participants had received a complete two-dose schedule of SARS-CoV-2 vaccines approximately 3–6 months prior to the first sample extraction and, out of them, 110 also received a booster dose within the same interval before the second sample extraction. Blood samples were collected at two time points, approximately between 3 and 6 months after the second dose and after the booster, to assess their sustained immunological response. The collected samples were processed by laboratory technicians and stored at -80ºC for subsequent serologic determinations. The samples were evaluated for titers of anti-S protein antibody (antiS-AB), as well as neutralizing antibodies (nAB) targeting wild-type SARS-CoV-2 and variants B.1.1.7 (Alpha), B.1.351 (Beta), B.1.617.2 (Delta), B.1.1.529 (Omicron), and P.1 (Gamma).

### Assessments

We collected various demographic and clinical information: from the subjects, including age, sex, type of vaccine received (BNT162b2 or mRNA-1237), dates of vaccination (second and third doses), dates of sample extraction, specific rheumatic disease, treatment regimen for the disease, and information on current glucocorticoid use as a binary variable.

Antibodies against S and N SARS-CoV-2 protein

Antibody response to SARS-CoV-2 S protein was measured using the Elecsys® Anti-SARS-CoV-2 S test (Roche Diagnostics International Ltd, Rotkreuz, Switzerland, quantitative) according to manufacture instructions. In these experiments, the standards and international units proposed by the WHO for the determination of antibodies against SARS-CoV-2 (Binding Antibody Units, BAU/ml) [22] were used. Based on previous studies [23], a value of 260 has been established as the minimum to consider the presence of a protective level against SARS-CoV-2 and, therefore, the minimum value to consider a patient as a responder to the vaccine.

### Neutralization assays

We employed the SARS-CoV-2 Variants Neutralizing Antibody 6-Plex ProcartaPlex™ Panel kit (Thermofisher Scientific, Waltham, MA, USA) in accordance with the manufacturer's instructions to determine nAB against several SARS-CoV-2 variants. The method involves a competitive assay between the ACE2 protein and the antibodies produced by the patient after vaccination and binding to the S protein of the several variants of SARS-CoV-2: wild type, B.1.1.529 (Omicron), B.1.1.7 (Alpha), B.1.351 (Beta), B.1.617.2 (Delta) and P.1 (Gamma). The Luminex® 200™ system (LuminexCorp, Austin, TX, USA) was used to evaluate the assay, which makes use of the Luminex xMAP technology. For result interpretation, we followed the manufacturer's recommended cutoff points, established after screening 160 healthy samples, to determine the vaccine response for each SARS-CoV-2 variant.

### Statistical methods

For descriptive purposes, categorical variables were presented as absolute and relative frequencies, while continuous measurements were described using medians, minimum and maximum values, and interquartile ranges. Univariate differences between subject groups were assessed by non-parametric techniques that included, Mann–Whitney tests (binary variables), Kruskal test (categorical variables with more than one category) and Fisher's tests for contingency tables. nAB were analysed as binary variables according to their positivity for immune response (see previous section).

For the analysis of antiS-AB, 95% confidence intervals (95%CI) for Fold-Changes (FC) between groups were computed using 1,000 bootstrap resamples stratified by the condition group. Multivariate associations were evaluated using a mixed-effects linear model. The fixed effects included sex, age, rheumatic condition, treatment type, use of glucocorticoids, type of vaccine, sample type (pre- or post-third dose), and time from the previous SARS-CoV-2 vaccine dose. Interactions between various factors were also included in the model, such as sex and sample type, treatment and sample type, rheumatic condition and sample type, use of glucocorticoids and sample type, time from second and third vaccine dose and sample type, glucocorticoids use and treatment regimen, and glucocorticoids use and rheumatic condition. Individual effects were considered as random effects in the model. When comparing treatment groups, only rheumatic conditions represented in the treatment groups involved in the comparisons were included in the analysis. Similarly, when analysing specific rheumatic conditions, only the treatment groups that included those conditions were considered. To fulfil the assumptions of the model, antibody quantifications were log2-transformed. Quantifications that did not reach the minimum detection threshold (threshold = 0.4) were assigned a value equal to half this threshold (antibody titre = 0.2). The adjusted group means at the original scale were retrieved from the models after undoing the log2 transformation. FCs and their 95%CI were obtained to express the magnitude of the effects. Statistical significance was determined using Wald tests derived from the models. The results were visually represented using a stripchart, which included the group means and 95%CI after adjustment for confounders. A significance level of 5% was used.

The predictive value of anti-S-AB titres and nAB positivity was evaluated using Receiver Operating Characteristic (ROC) analysis and the corresponding Area Under the Curve (AUC). Total accuracy, sensitivity, and specificity were determined for the optimal threshold, which was defined as the point on the ROC curve that is closest to the top-left corner (representing perfect sensitivity and specificity). Confidence intervals at a 95% confidence level were computed using bootstrap resampling. All the data analyses were performed using R [24].

## Results

### Patients description

We enrolled a cohort of 157 patients with various rheumatic conditions who had received a full two-dose regimen of mRNA-based SARS-CoV-2 vaccination and had no previous COVID-19 infection (Table 1). The rheumatic conditions represented in the study included Systemic Lupus Erythematosus (SLE, 22.3%), Rheumatoid Arthritis (RA, 22.9%), HLA-B27 positive Ankylosing Spondylitis (B27-AS, 14%), Psoriatic Arthritis (PSA, 25.5%), and Giant Cell Arteritis (GCA, 15.3%). Due to the specificities of each disease, there was heterogeneity in age and sex distributions across the groups. The proportion of women ranged from 32.5% in PSA to 89% in SLE, and patient age ranged from 52 years in B27-AS to 76 years in GCA (Table 1).

**Table 1:** Study patients' characteristics

		All *n* = 157	Systemic lupus erythematosus 35 (22.3%)	Rheumatoid arthritis 36 (22.9%)	B27-ankylosing spondylitis 22 (14.0%)	Psoriatic arthritis 40 (25.5%)	Giant cell arteritis 24 (15.3%)	*p* value
Sex—Female		100 (63.7%)	31 (88.6%)	30 (83.3%)	10 (45.5%)	13 (32.5%)	16 (66.7%)	< 0.0001
Age at third dose of SARS-CoV-2 vaccine		58.8 (33.5, 88.2)	53.3 (34.9, 83.0)	64.5 (47.4, 74.1)	51.9 (33.5, 75.5)	58.3 (39.4, 88.2)	75.6 (55.9, 83.9)	< 0.0001
Sample post-third SARS-CoV-2 provided		110 (70.1%)	20 (57.1%)	32 (88.9%)	17 (77.3%)	23 (57.5%)	18 (75.0%)	0.0105
Treatment type	Not treated or Glucorticoids only	25 (15.9%)	10 (28.6%)	0 (0.0%)	7 (31.8%)	0 (0.0%)	8 (33.3%)	< 0.0001
	Non-biological	48 (30.6%)	17 (48.6%)	8 (22.2%)	0 (0.0%)	16 (40.0%)	7 (29.2%)	
	Biological	69 (43.9%)	8 (22.9%)	18 (50.0%)	15 (68.2%)	19 (47.5%)	9 (37.5%)	
	JAK-inhibitors	15 (9.6%)	0 (0.0%)	10 (27.8%)	0 (0.0%)	5 (12.5%)	0 (0.0%)	
Glucocorticoids use		43 (27.4%)	7 (20.0%)	11 (30.6%)	0 (0.0%)	7 (17.5%)	18 (75.0%)	< 0.0001
mRNA-based vaccine type	mRNA-1273	100 (63.7%)	25 (71.4%)	30 (83.3%)	18 (81.8%)	25 (62.5%)	2 (8.3%)	< 0.0001
	BNT162b2	57 (36.3%)	10 (28.6%)	6 (16.7%)	4 (18.2%)	15 (37.5%)	22 (91.7%)	
2nd SARS-CoV-2 vaccine dose—baseline time interval (months)		5.3 (2.3, 7.6)	3.5 (2.3, 5.5)	5.3 (4.4, 7.0)	5.5 (3.4, 7.3)	5.4 (4.2, 7.0)	5.7 (4.9, 7.6)	< 0.0001
3rd SARS-CoV-2 vaccine dose—follow-up time interval (months)		3.8 (2.2, 7.0)	4.0 (2.8, 7.0)	3.6 (2.5, 7.0)	4.1 (2.7, 6.9)	3.5 (2.2, 5.4)	4.5 (2.9, 6.5)	0.1488
2nd to 3rd dose of the SARS-CoV-2 vaccine time interval (months)		6.7 (3.6, 11.1)	6.8 (3.6, 11.1)	6.7 (5.0, 8.5)	6.8 (5.3, 8.7)	6.8 (5.6, 10.0)	6.9 (5.9, 8.6)	0.2615

All patients underwent baseline sample collection prior to receiving the third dose of the SARS-CoV-2 vaccine. The median time interval between this baseline sample and the administration of the previous (second) dose was 5.3 months, although it was slightly shorter for SLE patients (3.5 months). In addition, a follow-up sample was obtained from 110 patients (70%) after the administration of the third dose. Loss of follow-up occurred due to patient mortality (3, 1.9%), COVID-19 infection (17, 10.8%), or patients not attending their sample extraction appointments (27, 17.2%). These patients showed half of the anti-S ABs average levels, lower presence of nABs for all variants analyzed, lower prevalence of SLE, higher frequency of RA, and similar distributions for the rest of parameters analyzed compared to subjects with both baseline and follow-up samples available (Additional file 2: Table S1).. The median time interval between the third dose and the follow-up sample was 3.8 months, with a range of 2.2 to 7.0 months across the entire series. The majority of patients in all disease groups (64% overall) were administered the mRNA-1273 vaccine, except for GCA patients who primarily received the BNT162b2 vaccine (92%) (Table 1).

The treatment regimens varied among the disease groups, reflecting differences in the current therapeutic approaches for these conditions. Approximately half of the patients with SLE (49%) and PSA (40%) were prescribed non-biological drugs, while none of the B27-AS patients received this type of treatment. In the B27-AS group, the predominant treatment prescription was biological agents (68%), which was also the therapy of choice for 50% of RA patients and 48% of PSA patients. JAK inhibitors (JAKi) were prescribed exclusively for patients with RA (28%) and PSA (13%). The use of glucocorticoids (GCs) also varied across disease groups, ranging from no B27-AS patients to 75% of GCA patients (Table 1).

### Anti-S protein antibodies levels prior to the third dose of the SARS-CoV-2 vaccine

**Fig. 1:**
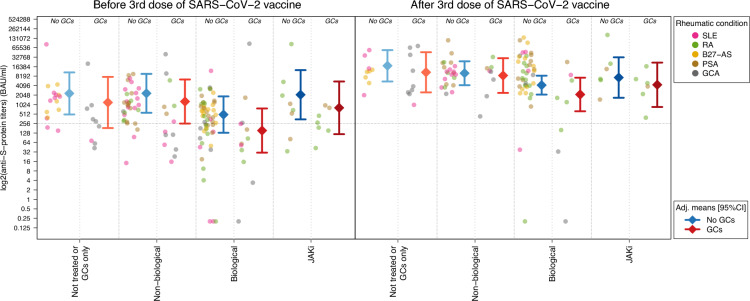
Anti-S protein antibodies titers by treatment groups before and after a third dose of mRNA-based SARS-CoV-2 vaccine. The circle-shaped dots show the levels of anti-S protein antibodies of the patients' baseline and follow-up samples. The diamond-shaped points and the adjacent segments display the adjusted group means of the titer values and their corresponding 95% confidence intervals, respectively. Dot colors indicate the patients’ disease condition. Adjusted means were derived from a mixed-effects linear model where sex, age, rheumatic condition, treatment type, use of glucocorticoids, type of vaccine, sample type (pre- or post-third dose) and time from previous dose where included as fixed effects, as well as the interaction between: sex and sample type; treatment and sample type; rheumatic condition and sample type; use of glucocorticoids and sample type; time from second and third vaccine dose and sample type; glucocorticoids use and treatment regimen; and glucorticoids use and rheumatic condition. Individual effects were included as random effects in this model. The *y*-axis is in log2-scale, while the value labels are displayed in the original scale of the titer values (BAU/ml). The horizontal dotted line indicates a value of 260 BAU/ml, which is typically used as a threshold to define a positive response to the vaccine. *SLE* Systemic Lupus Erythematosus, *RA* Rheumatoid Arthritis, *B27-AS* HLA-B27 positive Ankylosing Spondylitis, *PSA* Psoriatic Arthritis, *GCA* Giant Cell Arteritis, *GCs* Glucocorticoids; *JAKi* JAK inhibitors

Baseline titers of antiS-AB were significantly lower in patients receiving biological therapy compared to those who were not treated or were treated with GCs only (not-treated/GCs; 62% decrease, *p* value = 0.0132) and patients under non-biological drugs (66% decrease, *p* value = 0.038) (Fig. 1, Table 2 and Additional file 2: Table S2). These differences remained significant even after statistical control for potential confounders, (90% decrease and *p* value = 0.00007 compared to not-treated/GCs group; 71% decrease and *p* value = 0.0072 compared to the non-biological group; Table 3). There was also substantial variation among patients with different rheumatic conditions prior to the third SARS-CoV-2 vaccine dose, ranging from 239 (GCA) to 796 (PSA) BAU/ml (Additional file 2: Table S3). After adjusting for potential confounders, SLE patients displayed the lowest baseline antibody titers compared to other rheumatic conditions, with fold changes ranging from 3.21 (B27-AS, *p* value = 0.0850) to 8.27 (PSA, *p* value = 0.0027; Additional file 2: Table S4).

**Table 2:** Anti-S protein antibody levels and neutralizing antibodies positivity (nABs) by treatment group before (pre-third dose) and after (post-third dose) a third dose of the SARS-CoV-2 mRNA-based vaccine

	Pre-third dose					Post-third dose				
	Not-treated or GCs only *n* = 25	Non-biological *n* = 48	Biological *n* = 68	JAKi *n* = 15	*p* value	Not-treated or GCs only *n* = 18	Non-biological *n* = 30	Biological *n* = 50	JAKi *n* = 12	*p* value
Anti-S-protein antibody (BAU/ml)	761 [43, 83890]	857 [14, 40700]	288 [0, 87880]	534 [34, 85340]	0.0111	11445 [1002, 74400]	10850 [433, 112590]	7966 [0, 137780]	6374 [393, 166760]	0.8029
Wild type—Positive	19 (76.0%)	37 (77.1%)	37 (54.4%)	10 (66.7%)	0.0523	18 (100%)	30 (100%)	45 (90.0%)	12 (100%)	0.1561
B.1.1.529—Positive	4 (16.0%)	8 (16.7%)	3 (4.4%)	2 (13.3%)	0.09088	10 (55.6%)	18 (60.0%)	25 (50.0%)	7 (58.3%)	0.8489
B.1.1.7—Positive	11 (44.0%)	23 (47.9%)	19 (27.9%)	5 (33.3%)	0.1380	17 (94.4%)	29 (96.7%)	44 (88.0%)	10 (83.3%)	0.3733
B.1.351—Positive	10 (41.7%)	19 (39.6%)	12 (17.9%)	3 (20.0%)	0.0265	14 (87.5%)	29 (96.7%)	41 (83.7%)	10 (83.3%)	0.3002
B.1.617.2—Positive	11 (44.0%)	21 (43.8%)	16 (23.5%)	4 (26.7%)	0.0730	17 (94.4%)	29 (96.7%)	43 (86.0%)	10 (83.3%)	0.3146
P.1—Positive	10 (40.0%)	18 (37.5%)	15 (22.1%)	4 (26.7%)	0.1959	14 (77.8%)	28 (93.3%)	42 (84.0%)	10 (83.3%)	0.45344

Cells show the medians and ranges of anti-S protein antibody titers, and the frequencies and percentages of positivity for nABs of diferents SARS-CoV-2 variants including: Wild type, B.1.1.529 (Omicron), B.1.1.7 (Alpha), B.1.351 (Beta), B.1.617.2 (Delta) and P.1 (Gamma). The last row display the rheumatic conditions of patients represented in each treatment group and sample draw. *p* values are derived from a Kruskal–Wallis tests comparing treatment groups within each sample time point. JAKi: JAK inhibitors; GCs: Glucocorticoids

**Table 3:** Comparison of anti-S protein antibody titers between treatment groups within each time point of sample extraction

	Pre-third dose		Post-third dose	
	FC [95%CI]	*p* value	FC [95%CI]	*p* value
Non-biological—not treated or GCs only	0.62 [0.21, 1.90]	0.4059	0.42 [0.12, 1.50]	0.1845
Biological—not treated or GCs only	0.10 [0.03, 0.31]	0.00007	0.14 [0.04, 0.53]	0.0027
Biological—non-biological	0.29 [0.12, 0.72]	0.0072	0.49 [0.17, 1.43]	0.1904
JAKi—non-biological	0.50 [0.13, 1.90]	0.3066	0.37 [0.08, 1.66]	0.1935
JAKi—biological	3.21 [0.89, 11.55]	0.0742	1.15 [0.29, 4.59]	0.8483

Cells show Fold-changes (FC), 95% confidence intervals (CI) and I values of the corresponding comparison. FCs and *p* values were derived from a mixed-effects linear model where sex, age, rheumatic condition, treatment type, use of glucocorticoids, type of vaccine, sample type (pre- or post-3rd dose) and time from previous dose where included as fixed effects, as well as the interaction between: sex and sample type; treatment and sample type; rheumatic condition and sample type; use of glucocorticoids and sample type; time from second and third vaccine dose and sample type; glucocorticoids use and treatment regimen; and glucorticoids use and rheumatic condition. Individual effects were included as random effects in this model. *SLE* Systemic Lupus Erythematosus, *RA* Rheumatoid Arthritis, *B27-AS* HLA-B27 positive Ankylosing Spondylitis, *PSA* Psoriatic Arthritis, *GCA* Giant Cell Arteritis, *GCs* Glucocorticoids; *JAKi* JAK inhibitors, *GCs* Glucocorticoids, *FC* Fold-Change, *95%CI* 95% confidence interval

### Anti-S protein antibody levels after the third dose of the SARS-CoV-2 vaccine

After the administration of the third dose of the SARS-CoV-2 vaccine, patients' samples demonstrated elevated titers of antiS-AB compared to the pre-third dose samples (FC = 15.29, *p* value < 0.0001; Fig. 1). This increase was observed in roughly all patients in the cohort, regardless of their specific treatment regimen (Fold-Changes, FCs from 10.51 in the JAKi group to 22.35 for patients under biological therapy, *p* value < 0.03 in all cases; Fig. 1, Table 2, and Additional file 2: Table S5), as well as their rheumatic condition (FCs ranging from 18.07 in GCA patients to 23.30 in the SLE group, *p* value < 0.04 in all cases; Additional file 1: Figure S1 and Additional file 2: Tables S3 and S6). After control for potential confounders, the magnitude of this titer's raise was not statistically significant across groups defined by the specific rheumatic disease or by their prescribed therapy (interaction *p* values = 0.5300 and 0.3059, respectively). However, it is noteworthy that this increase was notably higher in men (Fold Change, FC = 27.13) compared to women (FC = 8.62, interaction *p* value = 0.0095).

Similarly, to the baseline samples, post-third dose levels in the Biological group were significantly lower compared to the not-treated/GCs group, even after control for confounders (86% decrease, *p* value = 0.0027; Table 3). Only five patients (5%) reached a value under 260 BAU/ml after the third dose, a threshold typically used to define a positive response to the vaccine and, notably, all of them were under biological therapy (Fig. 1). After the third dose, the differences observed in baseline for SLE compared to the rest of rheumatic conditions became less pronounced (FCs ranging from 1.46 to 4.30) and lost their statistical significance (Additional file 2: Table S4).

### Glucocorticoids and anti-S protein antibody levels

Pre-third dose antibody levels were consistently lower in patients receiving glucocorticoids across all treatment groups, with decreases ranging from 83% (Biological) to 69% (JAKi) (Additional file 2: Table S7). These decreases were statistically significant for untreated patients (80% decrease, *p* value = 0.0272) and those receiving biological therapy (83% decrease, *p* value = 0.0402). The differences in post-third dose samples became less pronounced except for patients treated with biological drugs, where GCs users still exhibited a statistically significant 87% decrease in antiS-AB titers compared to non-users (*p* value = 0.0124; Additional file 2: Table S7). None of these differences remained statistical significant after controlling for potential confounders, although they still showed considerable magnitude in some cases (Table 4). Overall, the use of GCs was associated with a 58% decrease in baseline antiS-AB levels and a 36% decrease after the administration of the third SARS-CoV-2 vaccine dose, although these differences did not reach statistical significance (*p* values 0.0577 and 0.2931, respectively; Table 4).

**Table 4:** Comparison of anti-S protein antibody titers between users and non-users of glucocroticoids (GCs) within each treatment group and time point of sample extraction

	Pre-third dose		Post-third dose	
	FC [95%CI]	*p* value	FC [95%CI]	*p* value
Overall	0.42 [0.22, 1.21]	0.0577	0.64 [0.22, 1.84]	0.4079
Non-biological	0.56 [0.17, 1.03]	0.3903	0.80 [0.19, 3.38]	0.7636
Biological	0.33 [0.10, 1.13]	0.0775	0.47 [0.12, 1.80]	0.2706
JAKi	0.38 [0.06, 2.56]	0.3180	0.54 [0.07, 3.91]	0.5418

Cells show Fold-changes (FC), 95% confidence intervals (CI) and *p* values of the corresponding comparison. FCs and *p* values were derived from a mixed-effects linear model where sex, age, rheumatic condition, treatment type, use of glucocorticoids, type of vaccine, sample type (pre- or post-third dose) and time from previous dose where included as fixed effects, as well as the interaction between: sex and sample type; treatment and sample type; rheumatic condition and sample type; use of glucocorticoids and sample type; time from second and third vaccine dose and sample type; glucocorticoids use and treatment regimen; and glucorticoids use and rheumatic condition. Individual effects were included as random effects in this model. *SLE* Systemic Lupus Erythematosus, *RA* Rheumatoid Arthritis, *B27-AS* HLA-B27 positive Ankylosing Spondylitis, *PSA* Psoriatic Arthritis, *GCA* Giant Cell Arteritis, *GCs* Glucocorticoids; *JAKi* JAK inhibitors, *GCs* Glucocorticoids, *FC* Fold-Change, *95%CI* 95% confidence interval

Regarding rheumatic conditions, up to 75% decrease in baseline antiS-AB levels was observed in GCs users after adjusting for confounders (25% decrease in SLE and GCA patients; Additional file 2: Table S8). However, these differences were not statistically significant (*p* values 0.0922 and 0.1515, respectively) and their magnitude substantially diminished after the administration of the third dose of the SARS-CoV-2 vaccine (Additional file 2: Table S8).

### Neutralizing antibodies for SARS-CoV-2 variants

As expected, neutralizing antibodies (nAB) against the wild-type strain exhibited the highest rates of positivity before and after the administration of the third dose (50% and 93%, respectively; *p* value < 0.002 for all variants). In contrast, the B.1.1.529 variant (Omicron) showed the lowest positivity across all patient groups (11% and 55%, respectively; all *p* values < 0.002). Notably, among the 27 patients who experienced a COVID-19 infection between the second and third vaccine administration, none of them demonstrated evidence of neutralizing antibodies against the B.1.1.529 variant, which was epidemiologically predominant during the study period. The variants B.1.1.7 (Alpha), B.1.351 (Beta), B.1.617.2 (Delta), and P.1 (Gamma) displayed similar levels of positivity, approximately 30% before the third dose and 90% after the third dose.

**Fig. 2:**
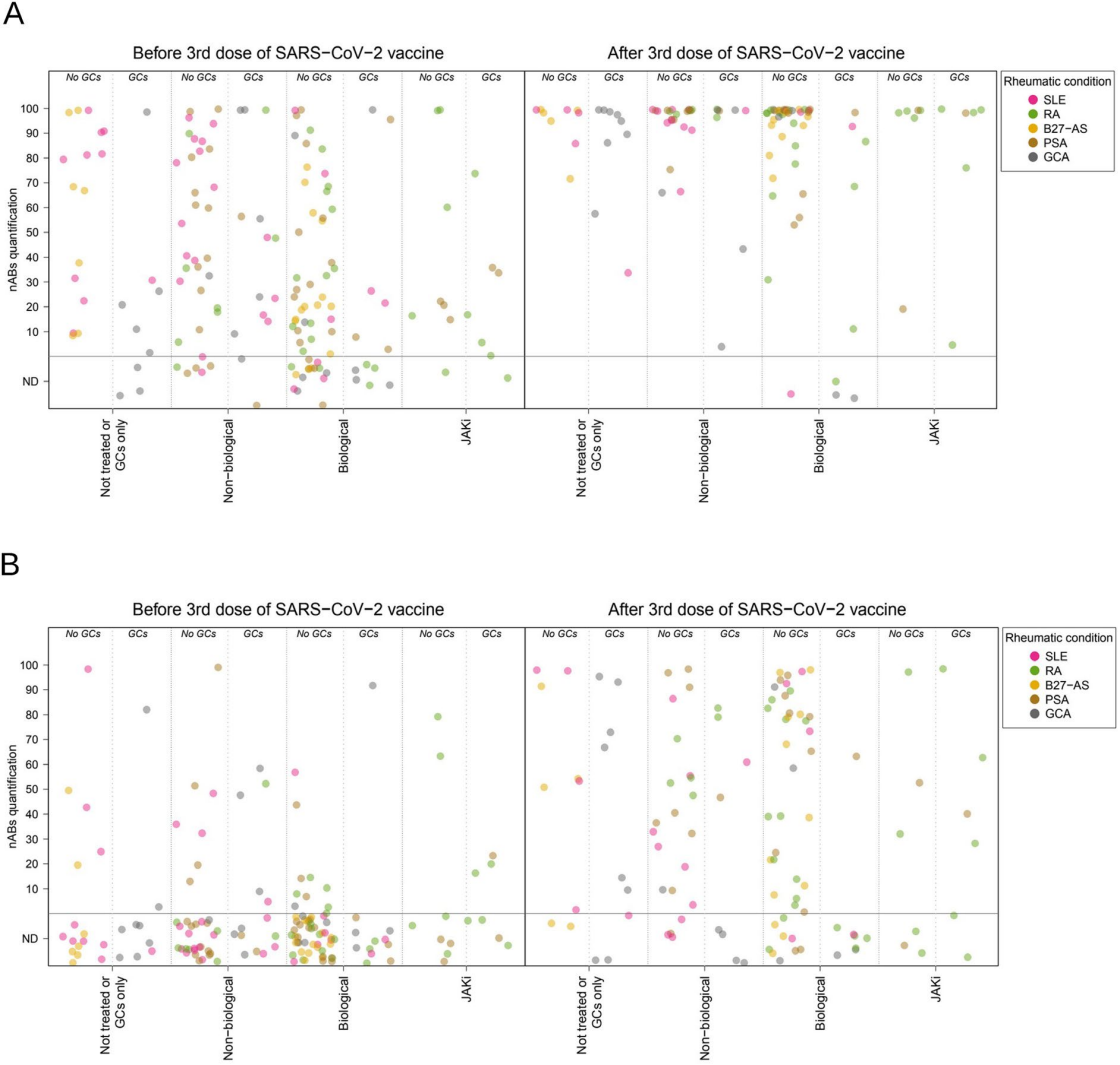
Quantification of neutralizing antibodies by treatment regimen before and after a third dose of mRNA-based SARS-CoV-2 vaccine for variants Wild type (**A**) and B.1.1.529 (Omicron) (**B**). The dots show the quantitative estimations of neutralizing antibodies abundance of the patients' baseline and follow-up samples. Dot colors indicate the patients' rheumatic condition. Horizontal dotted lines display the threshold for no detection of antibody (ND) and positivity. *SLE* Systemic Lupus Erythematosus, *RA* Rheumatoid Arthritis, *B27-AS* HLA-B27 positive Ankylosing Spondylitis, *PSA* Psoriatic Arthritis, *GCA* Giant Cell Arteritis, *GCs* Glucocorticoids; *JAKi* JAK inhibitors, *nABs* neutralizing antibodies

Despite overall differences in positivity, all variants demonstrated a consistent pattern across treatment groups, similar to that observed for antiS-AB titers (Table 2, Fig. 2, and Additional file 1: Figures S2 to S4). This pattern included a lower proportion of positivity at baseline among patients receiving biological therapy (54% for wild type, 4% for B.1.1.529, and 18–28% for other variants) or JAK inhibitors (67%, 13% and 20–33%, respectively) compared to those receiving non-biological drugs (77%, 17% and 38–48%, respectively) or no treatment other than glucocorticoids (76%, 16% and 40–44%, respectively).

Following the administration of the third dose of the SARS-CoV-2 vaccine, the positivity for variants B.1.1.7, B.1.351, B.1.617.2, and P.1 increased to near 90% in untreated patients (79–94%) and the non-biological group (93–97%). However, post-third dose positivity was slightly lower in patients under biological drugs (84–86%) or JAK inhibitors (83.3%) for these variants. This lower positivity in the biological group after the third dose was also observed for the wild-type strain (90% vs. 100% in the other treatment groups) and the B.1.1.529 variant (50% vs. 56–60%). However, differences in the nAB response across treatment groups were only statistically significant for variant B.1.351 in the pre-third dose sample (*p* value = 0.0265) (Table 2). No statistically significant differences in the positivity of any variant were found across groups of patients defined by their rheumatic condition after the administration of the third vaccine dose (Additional file 2: Table S3).

The presence of neutralizing antibodies (nABs) also showed a similar trend to that observed for antiS-AB in relation to glucocorticoid (GCs) prescription. GCs users had a lower frequency of nAB positivity, particularly after receiving the third dose of the SARS-CoV-2 vaccine (Additional file 2: Table S9). Statistically significant decreases in nAB positivity were observed for variants B.1.1.7 (19%, *p* value = 0.0050), B.1.351 (19%, *p* value = 0.00097), and B.1617.2 (18%, *p* value = 0.0109) in the post-third dose sample (Additional file 2: Table S9). These decreases were even more pronounced in patients treated with biological drugs, where GCs users experienced a decrease ranging from 44.2% to 55.4% (*p* values < 0.05 in all cases; Additional file 2: Table S10).

Finally, there was good concordance between the levels of nABs and antiS-AB, as evidenced by the AUC values measuring the ability of antiS-AB titres to predict variant positivity. These AUC values ranged from 0.896 (wild-type strain in pre-third dose samples) to 0.997 (variant B.1.1.7 in the post-third dose samples), with sensitivities ranging from 78% (wild type in pre-third dose) to 99% (B.1.617.2 in post-third dose), and specificities from 75% (P.1 in post-third dose) to 100% (wild type, B.1.1.7 and B.1.351 in post-third dose) (Additional file 1: Figures S5 and S6).

## Discussion

The present study aimed to investigate the medium-term effects of SARS-CoV-2 vaccination (3–6 months) after the second and third vaccine doses in patients with AIRD. For doing so, we employed a cohort of 157 AIRD patients who had received a full two-dose regimen of mRNA-based SARS-CoV-2 vaccination and had no previous COVID-19 infection. Our primary objectives were to assess the immune response following vaccination, examine the impact of different treatment regimens on this response, and analyze the presence of nAB against various variants of the SARS-CoV-2. Our data revealed three significant findings. First, we observed a notable enhancement in the immune response through the implementation of a booster strategy in the majority of AIRD patients, regardless of their specific rheumatic condition or therapeutic option. Secondly, we identified a negative impact on the vaccine response among patients undergoing biological treatment compared to those receiving other therapeutic options. Finally, we found that patients prescribed with GCs exhibited a diminished response to the vaccine in comparison to non-GCs users, especially in patients under biological therapy.

We observed a notable improvement in immune induction after administering a booster dose to patients with AIRD, including those who had a limited response following the second dose. This improvement was evident in the overall increase in levels of both antiS-ABs (FC = 15.29) and nABs against different variants of the SARS-CoV-2 (from 43% to 59%) and, of note, it was higher in men compared to women. Previous works have consistently shown that this enhanced immunological response is associated with a lower incidence of severe SARS-CoV-2 infections and hospital admissions in AIRD patients who received a triple-vaccination regimen, compared to those who received only two doses or remained unvaccinated [21]. Other studies focusing on patients with AIRD, such as rheumatoid arthritis (RA), ankylosing spondylitis (AS), and psoriatic arthritis (PsA), have demonstrated an improved immunological response four weeks after administering a booster vaccine scheme [25, 26]. It is worth noting that, in these works, patients initially exhibited lower levels of nABs compared to healthy individuals [27]. However, following the booster dose, AIRD patients experienced a more significant increase in their nAB levels compared to the control group. Our data align with these previous observations and demonstrate a sustained immunological response in AIRD patients following the booster vaccination, as indicated by the increase in antiS-ABs and nABs before and after the third vaccination dose. These findings emphasize the effectiveness of booster doses in enhancing the immune response and generating a sustained immunological defense against SARS-CoV-2 in patients with AIRD.

Second, it was observed that patients undergoing biological treatment exhibited a lower level of immunological protection to SARS-CoV-2, and a higher proportion of these patients remained below the protective threshold both for antiS-ABs and nABs, even following the administration of a booster. Previous studies have reported that, in general, most patients with AIRD regained a humoral response six weeks after receiving the booster dose, except for those undergoing rituximab treatment [19]. However, there is ongoing controversy surrounding the specific biological and immunosuppressive agents that have the most negative impact on the immune response. Among the different treatment regimens, rituximab appears to exert the most negative effect on this response [20]. Previous studies have also shown that biological drugs, in general, are associated with higher risks of lower immunological response rates [27, 28], with notable exceptions such as IL17 inhibitors, which do not seem to produce this undesirable effect [29]. Among non-biological immunosuppressive drugs, salazopyrin has showed no impact in the immune response against the SARS-CoV-2, while mycophenolate clearly impacts the level of the immunological protection. There are uncertainties regarding the impact of other treatments, which may be influenced by factors such as age and concomitant conditions [29, 30]. A recent large cohort study involving patients with various AIRD, predominantly rheumatoid arthritis (RA), and receiving multiple immunomodulatory treatment regimens, found that compared to antimalarials, anti-CD20 monoclonal antibodies, CTLA-4 Ig, mycophenolate, IL6 inhibitors, JAK inhibitors, and TNF inhibitors showed adjusted hazard ratios ranging from 5.20 to 1.70, indicating a compromised immune response to the SARS-CoV-2 vaccine [31]. While CD20 inhibitors may potentially benefit from new passive immunity or vaccines, other biologics and certain immunomodulators or immunosuppressive drugs like mycophenolate require careful monitoring of the immune response to make informed decisions regarding the best strategies for these patients, taking into account concerns about long-term efficacy [32]. In addition, a recent study reported an accelerated decline in the immunological response among AIRD patients after receiving the third dose of the SARS-CoV-2 vaccine, highlighting the importance of diligent follow-up to enhance the protective strategy [33]. Unfortunately, our study's sample size did not allow for specific comparisons between drugs within each treatment group, thus unable to contribute to this controversy.

The findings of our study also provide relevant insights into the impact of GC use on the immunological response to SARS-CoV-2 vaccination in AIRD patients. Consistent with a previous work of our group [16], we observed a significant decrease in baseline antiS-AB levels in patients receiving GCs, indicating a compromised immune response in the mid-term after the second dose of the vaccine. This decrease ranged from 69% (JAKi) to up to 83% in patients receiving biological therapy and, although it became less pronounced after the administration of the third vaccine dose, it remained statistically significant for patients treated with biological drugs. In the overall series, we observed a statistically significant 64% decrease in antiS-AB levels associated with GCs before the administration of the third vaccine dose, even after controlling for potential confounders. Regarding specific rheumatic conditions, it is worth noting that we observed a 75% decrease in antiS-AB titers in SLE patients which, although not statistically significant in our present data, is consistent with our previous work [16]. The impact of GCs on nABs was also evident, as the frequency of positive responses to the vaccines was significantly lower in GCs users even in post-third dose samples (16–19%), where this decrease was statistically significant for three out of the five variants assessed. Notably, patients treated with biological drugs exhibited more pronounced declines, with reductions in nAB positivity ranging from 44% to 55%. These findings underscore the importance of further research in larger cohorts to better understand the complex interplay between GC use and the immunological response to SARS-CoV-2 vaccination in AIRD patients.

Compared to other SARS-CoV-2 variants, the proportion of vaccine responders analyzed for nABs against the B.1.1.529 (Omicron) was comparatively lower, indicating a higher number of patients below the protection threshold. In addition, the improvement in antibody levels against the Omicron variant after the booster administration was less pronounced compared to other variants. These findings align with previous studies that have reported the potential immune evasion of the Omicron variant in AIRD patients, highlighting the need for novel strategies in their immunization [34]. Subsequent studies in AIRD patients have confirmed these findings, demonstrating a diminished immunological response specifically to the Omicron variant [27]. Moreover, a previous study conducted among vaccinated AIRD patients reported a higher incidence of breakthrough infections during the Omicron wave compared to previous rates [35]. It is noteworthy that the risk of contracting the Omicron variant was found to be lower in patients with hybrid immunization (those who had a previous active COVID-19 infection) compared to fully vaccinated patients. Among the fully vaccinated patients, a 17% rate of breakthrough infections was observed during the Omicron era [36]. Importantly, in our dataset, all 17 patients who experienced breakthrough infections between the two sample collections exhibited Omicron nAB levels below the predetermined protection threshold, indicating a lack of immunological response in these individuals. In light of these emerging SARS-CoV-2 variants, there is a pressing need for ongoing research to enhance vaccine effectiveness and explore strategies such as monoclonal antiviral treatments [37], to ensure adequate protection against SARS-CoV-2 for AIRD patients, who may be particularly vulnerable to the evolving landscape of viral variants.

The present study has several limitations inherent to its observational nature conducted in a clinical practice setting, which involved patients with diverse autoimmune-mediated rheumatic diseases and various treatment regimens. Some of the treatments considered were specific to rheumatic conditions to some extent, which made it challenging to statistically control for potential confounders and discern between disease and therapy effects. This complexity impaired the effective sample size in certain comparisons and may influence the interpretation of the results. In addition, the diversity in treatment regimens and the relatively small number of patients enrolled restricted our ability to conduct a comprehensive comparison between specific drugs to identify differences in immune responses among different biological agents (Additional file 2: Table S11).

Roughly 30% of patients did not undergo a post-third dose sample due to death (2%), SARS-CoV-2 infection (11%) or not attending the extraction appointment (17%). Overall, these patients showed lower levels of anti-S ABs and a lower presence of nABs for all variants analyzed, which might indicate a poorer overall health status. However, they also showed largely similar characteristics to those contributing with two samples to the study. In addition, the magnitude of the anti-S antibody titer raises after the third vaccine dose did not differ significantly across groups defined by the specific rheumatic disease or by their prescribed therapy. These observations suggest a minimal impact of follow-up losses on the conclusions of our study. Another limitation of our study is that, while our analysis focused on the specific therapies administered for rheumatic conditions, we did not account for other medications that patients might be taking, and which could potentially influence vaccine response.

Despite these limitations, our findings suggest that treatment regimens have a greater influence on the decline of the immunological response rather than the underlying disease itself. To further confirm these findings, future studies should aim to address these limitations by including a larger sample size, accounting for a wider range of AIRD conditions, and standardizing treatment regimens. On the other hand, a notable strength of our study was the evaluation conducted at approximately 3–6 months following the administration of the second and third vaccine doses. This extended timeframe allowed us to assess the sustainability of the immunological response in patients with AIRD and, hence, the durability of the immune protection conferred by the SARS-CoV-2 vaccination in this population.

In conclusion, our study demonstrates the effectiveness of booster doses in enhancing the immune response and generating a sustained immunological defense against SARS-CoV-2 in patients AIRD, as indicated by increased serum levels of antiS-ABs and nABs. However, our findings suggest that patients under biological therapy or GC treatment may experience an impaired immune response to the vaccine, potentially increasing their risk of COVID-19 infection and disease severity. The emergence of divergent virus variants emphasizes the need for ongoing research to enhance vaccine effectiveness and explore alternative strategies to ensure adequate protection for AIRD patients. Future studies should focus on optimizing vaccine response in these vulnerable populations and identifying strategies to address the challenges posed by evolving virus variants.

### Supplementary Information

**Additional file 1:  Figure S1**. Anti-S protein antibodies titers by rheumatic disease groups before and after a third dose of mRNA-based SARSCoV-2 vaccine.** Figure S2**. Quantification of neutralizing antibodies for B.1.1.7 (Alpha) variant of SARS-CoV-2 by treatment regimen before and after a third dose of mRNA-based SARS-CoV-2 vaccine.** Figure S3**. Quantification of neutralizing antibodies for B.1.351 (Beta) variant of SARS-CoV-2 by treatment regimen before and after a third dose of mRNA-based SARS-CoV-2 vaccine.** Figure S4**. Quantification of neutralizing antibodies for B.1.617.2 (Delta) variant of SARS-CoV-2 by treatment regimen before and after a third dose of mRNA-based SARS-CoV-2 vaccine.** Figure S5**. Concordance of anti-S protein and neutralizing antibodies in pre-third dose samples.** Figure S6**. Concordance of anti-S protein and neutralizing antibodies in post-third dose samples.

**Additional file 2:  Table S1**. Patients' characteristics by sample availability for analysis.** Table S2**. Fold-changes (FC), boostrap 95% confidence intervals (CI) and p-values for comparison of treatment groups within each time point of sample extraction. P-values are derived from a Mann-Whitney's test. JAKi: JAK inhibitors; GCs: Glucocorticoids.** Table S3**. Anti-S protein antibody levels and neutralizing antibodies positivity (nABs) by rhuematic condition group before (Pre-3rd dose) and after (Post-3rd dose) of a third dose of the SARS-CoV-2 mRNA-based vaccine.** Table S4**. Comparison of anti-S protein antibody titers between rheumatic condition groups within each time point of sample extraction.** Table S5**. Increase of anti-S protein antibody levels after the third dose of the SARS-CoV-2 vaccine by treatment group.** Table S6**. Increase of anti-S protein antibody levels after the third dose of the SARS-CoV-2 vaccine by rheumatic condition.** Table S7**. Anti-S protein antibody levels by treatment group before (Pre-3rd dose) and after (Post-3rd dose) a third dose of the SARS-CoV-2 mRNA-based vaccine.** Table S8** Comparison of anti-S protein antibody titers between users and non-users of Glucocroticoids (GCs) within each rheumatic condition and time point of sample extraction. Cells show Fold-changes (FC), 95% confidence intervals (CI) and p-values of the corresponding comparison.** Table S9**. Positivity of neutralizing antibodies by glucorcorticoids use in the overall series.** Table S10**. Positivity of neutralizing antibodies by glucorcorticoids use in patients under biological treatment.** Table S11**. Distribution of biological treatments by use of Glucocorticoids (GCs).

## Data Availability

The data sets used and analysed during the current study are available from the corresponding author on reasonable request.

## Abbreviations

AIRD: Autoimmune Inflammatory Rheumatic Diseases
antiS-AB: Antibodies against the Spike protein
AUC: Area Under the Curve
SpA: Axial Spondylitis B27 +
BAU/ml: Binding Antibody Units
CI: Confidence intervals
FC: Fold-Changes
GCs: Glucocorticoids
GCA: Giant Cell Arteritis
nAB: Neutralizing antibodies
PsA: Psoriatic Arthritis
SLE: Systemic Lupus Erythematosus
RA: Rheumatoid Arthritis
ROC: Receiver Operating Characteristic
