# Supplementary material for: Biological and glucocorticoids treatment impair the medium-term immunogenicity to SARS-CoV-2 mRNA vaccines in autoimmune inflammatory rheumatic diseases
Source: Eur J Med Res. 2024 Jan 5;29:28. doi: 10.1186/s40001-023-01620-7 (PMC10768211; doi:10.1186/s40001-023-01620-7)
Supplement: Supplementary file 1 — Additional file 1: Figure S1. Anti-S protein antibodies titers by rheumatic disease groups before and after a third dose of mRNA-based SARSCoV-2 vaccine. Figure S2. Quantification of neutralizing antibodies for B.1.1.7 (Alpha) variant of SARS-CoV-2 by treatment regimen before and after a third dose of mRNA-based SARS-CoV-2 vaccine. Figure S3. Quantification of neutralizing antibodies for B.1.351 (Beta) variant of SARS-CoV-2 by treatment regimen before and after a third dose of mRNA-based SARS-CoV-2 vaccine. Figure S4. Quantification of neutralizing antibodies for B.1.617.2 (Delta) variant of SARS-CoV-2 by treatment regimen before and after a third dose of mRNA-based SARS-CoV-2 vaccine. Figure S5. Concordance of anti-S protein and neutralizing antibodies in pre-third dose samples. Figure S6. Concordance of anti-S protein and neutralizing antibodies in post-third dose samples. [file 40001_2023_1620_MOESM1_ESM.pdf]

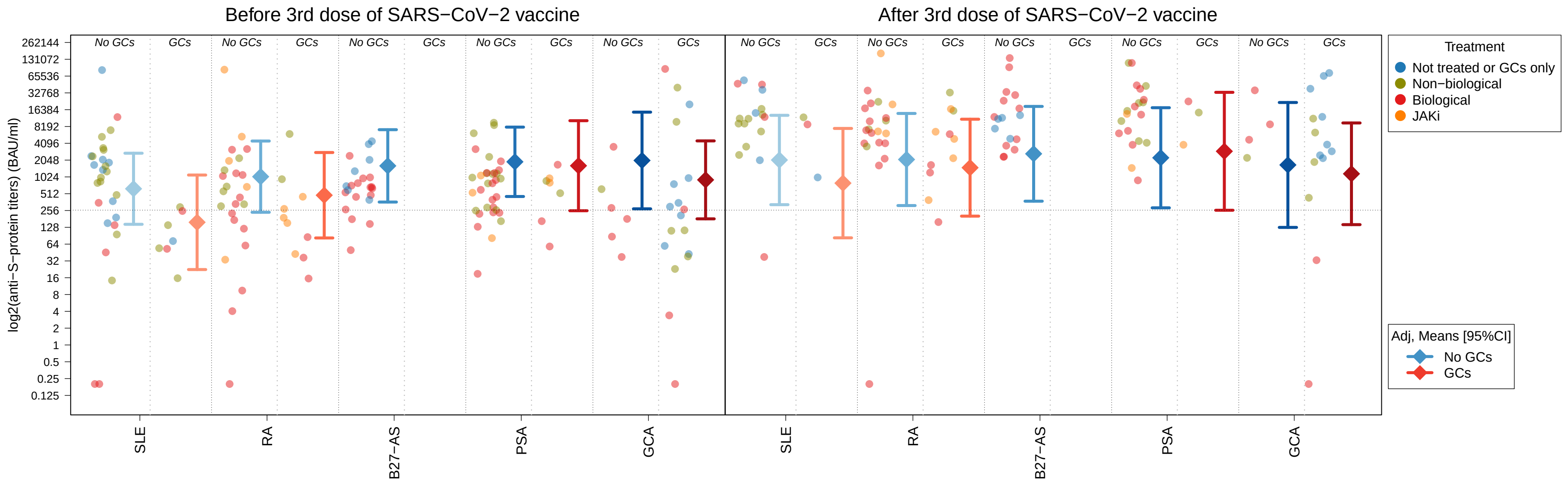

Suplementary Figure S1. Anti-S protein antibodies titers by rheumatic disease groups before and after a third dose of mRNA-based SARS-CoV-2 vaccine. The circle-shaped dots show the levels of anti-S protein antibodies of the patients' baseline and follow-up samples. The diamond-shaped points and the adjacent segments display the adjusted group means of the titer valuues and their corresponding 95% confidence intervals, respectively. Dot colors indicate the patients' treatment regimen. Adjusted means were derived from a mixed-effects linear model where sex, age, rheumatic condition, treatment type, use of glucocorticoids, type of vaccine, sample type (pre- or post-3rd dose) and time from previous dose were included as fixed effects, as well as the interaction between: sex and sample type; treatment and sample type; rheumatic condition and sample type; use of glucocorticoids and sample type; time from 2nd and third vaccine dose and sample type; glucocorticoids use and treatment regimen; and glucorticoids use and rheumatic condition. Individual effects were included as random effects in this model. The y-axis is in log2-scale, while the value labels are displayed in the original scale of the titer values (BAU/ml), The horizontal dotted line indicates a value of 260 BAU/ml, which is typically used as a threshold to define a positive response to the vaccine. **SLE:** Systemic Lupus Erythematosus; **RA:** Rheumatoid Arthritis; **B27-AS:** HLA-B27 positive Ankylosing Spondylitis; **PSA:** Psoriatic Arthritis; **GCA:** Giant Cell Arteritis; **GCs:** Glucocorticoids; **JAKi:** JAK inhibitors.

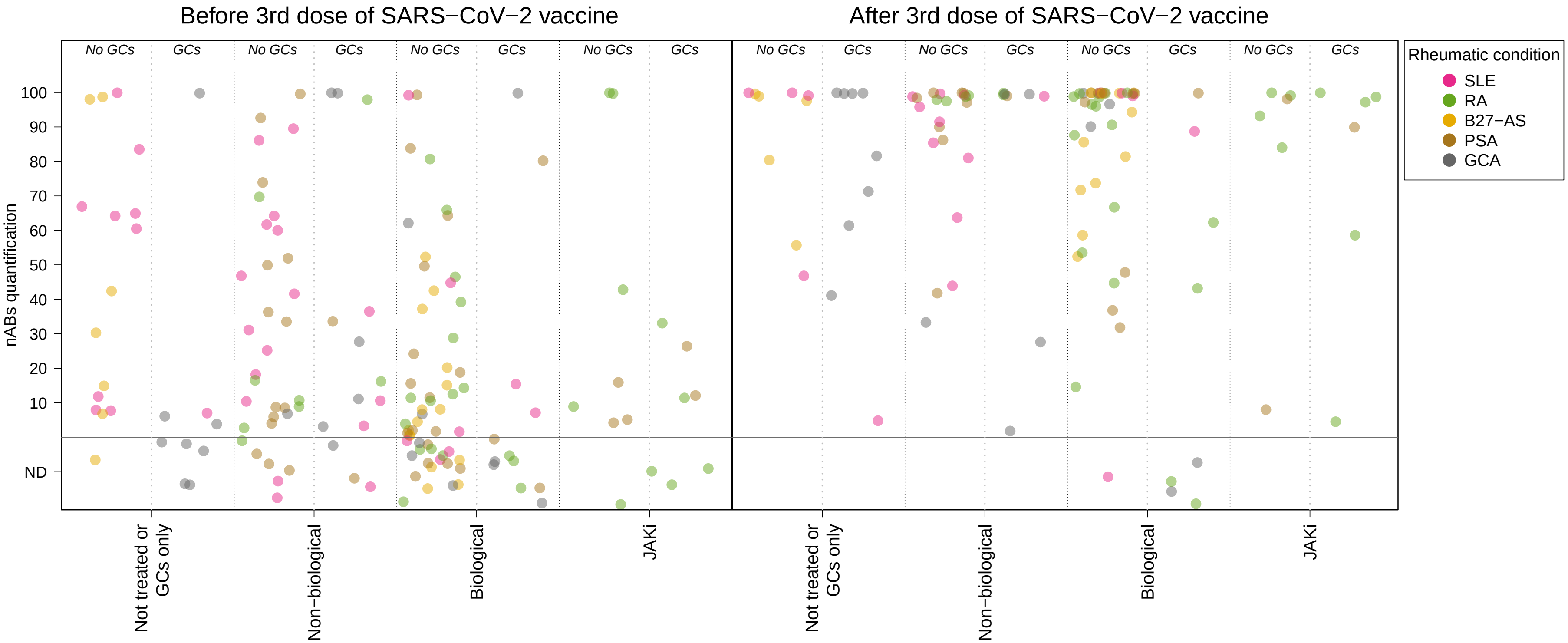

Suplementary Figure S2. Quantification of neutralizing antibodies for **B.1.1.7** (Alpha) variant of SARS-CoV-2 by treatment regimen before and after a third dose of mRNA-based SARS-CoV-2 vaccine. The dots show the quantitative estimations of neutralizing antibodies abundance of the patients' baseline and follow-up samples. Dot colors indicate the patients' rheumatic condition. Horizontal dotted lines display the threshold for no detection of antibody (**ND**) and positivity. **SLE**: Systemic Lupus Erythematosus; **RA:** Rheumatoid Arthritis; **B27-AS:** HLA-B27 positive Ankylosing Spondylitis; **PSA:** Psoriatic Arthritis; **GCA:** Giant Cell Arteritis; **GCs:** Glucocorticoids; **JAKi:** JAK inhibitors; **nABs:** neutralizing antibodies.

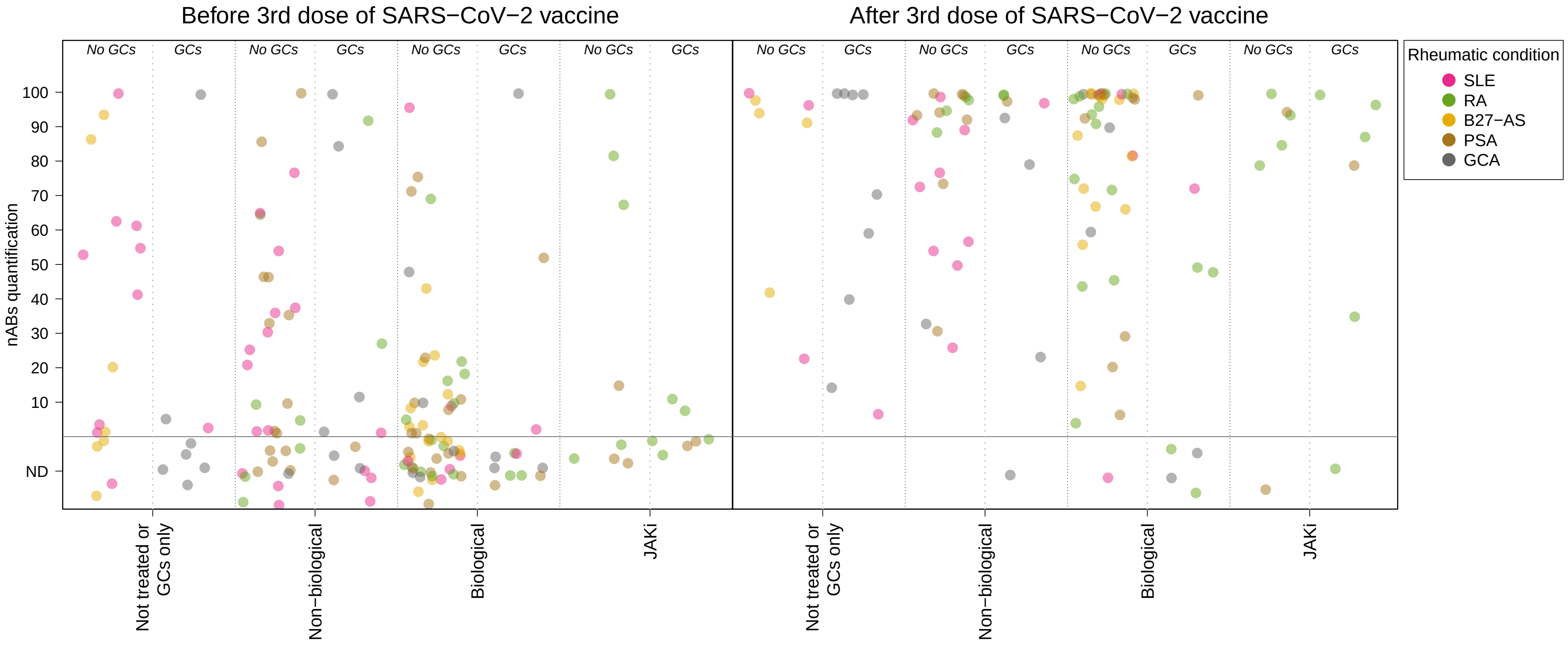

Suplementary Figure S3. Quantification of neutralizing antibodies for **B.1.351** (Beta) variant of SARS-CoV-2 by treatment regimen before and after a third dose of mRNA-based SARS-CoV-2 vaccine. The dots show the quantitative estimations of neutralizing antibodies abundance of the patients' baseline and follow-up samples. Dot colors indicate the patients' rheumatic condition. Horizontal dotted lines display the threshold for no detection of antibody (**ND**) and positivity. **SLE**: Systemic Lupus Erythematosus; **RA:** Rheumatoid Arthritis; **B27-AS:** HLA-B27 positive Ankylosing Spondylitis; **PSA:** Psoriatic Arthritis; **GCA:** Giant Cell Arteritis; **GCs:** Glucocorticoids; **JAKi:** JAK inhibitors; **nABs:** neutralizing antibodies.

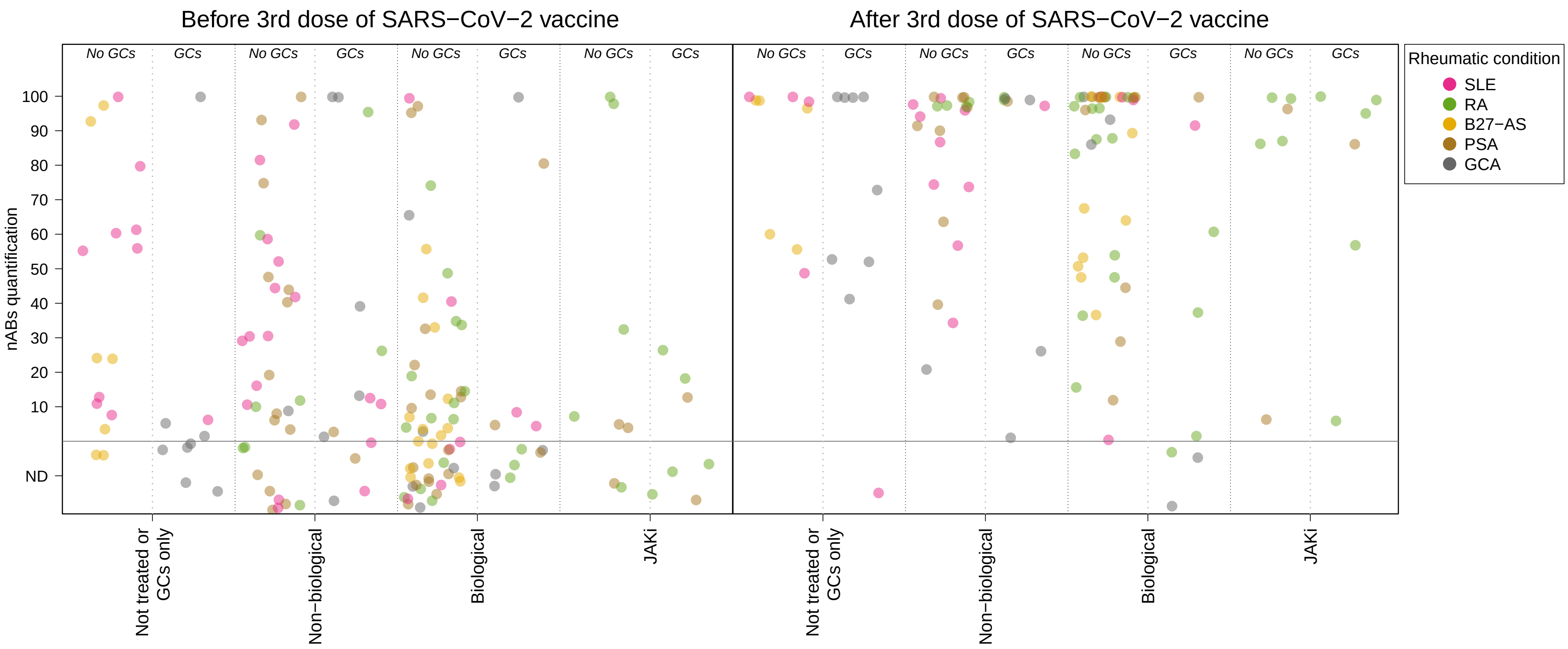

Suplementary Figure S4. Quantification of neutralizing antibodies for **B.1.617.2** (Delta) variant of SARS-CoV-2 by treatment regimen before and after a third dose of mRNA-based SARS-CoV-2 vaccine. The dots show the quantitative estimations of neutralizing antibodies abundance of the patients' baseline and follow-up samples. Dot colors indicate the patients' rheumatic condition. Horizontal dotted lines display the threshold for no detection of antibody (**ND**) and positivity. **SLE**: Systemic Lupus Erythematosus; **RA:** Rheumatoid Arthritis; **B27-AS:** HLA-B27 positive Ankylosing Spondylitis; **PSA:** Psoriatic Arthritis; **GCA:** Giant Cell Arteritis; **GCs:** Glucocorticoids; **JAKi:** JAK inhibitors; **nABs:** neutralizing antibodies.

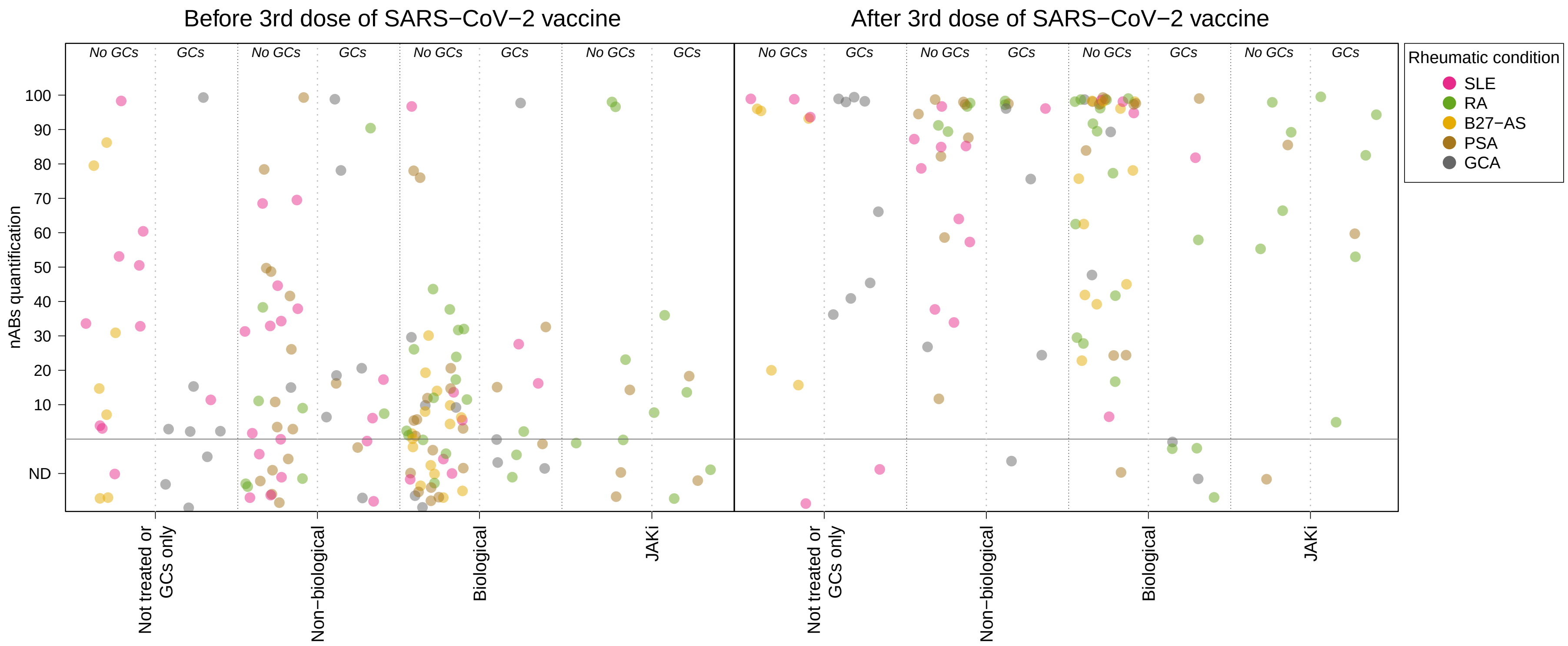

Suplementary Figure S4. Quantification of neutralizing antibodies for **P.1** (Gamma) variant of SARS-CoV-2 by treatment regimen before and after a third dose of mRNA-based SARS-CoV-2 vaccine. The dots show the quantitative estimations of neutralizing antibodies abundance of the patients' baseline and follow-up samples. Dot colors indicate the patients' rheumatic condition. Horizontal dotted lines display the threshold for no detection of antibody (**ND**) and positivity. **SLE**: Systemic Lupus Erythematosus; **RA:** Rheumatoid Arthritis; **B27-AS:** HLA-B27 positive Ankylosing Spondylitis; **PSA:** Psoriatic Arthritis; **GCA:** Giant Cell Arteritis; **GCs:** Glucocorticoids; **JAKi:** JAK inhibitors; **nABs:** neutralizing antibodies.

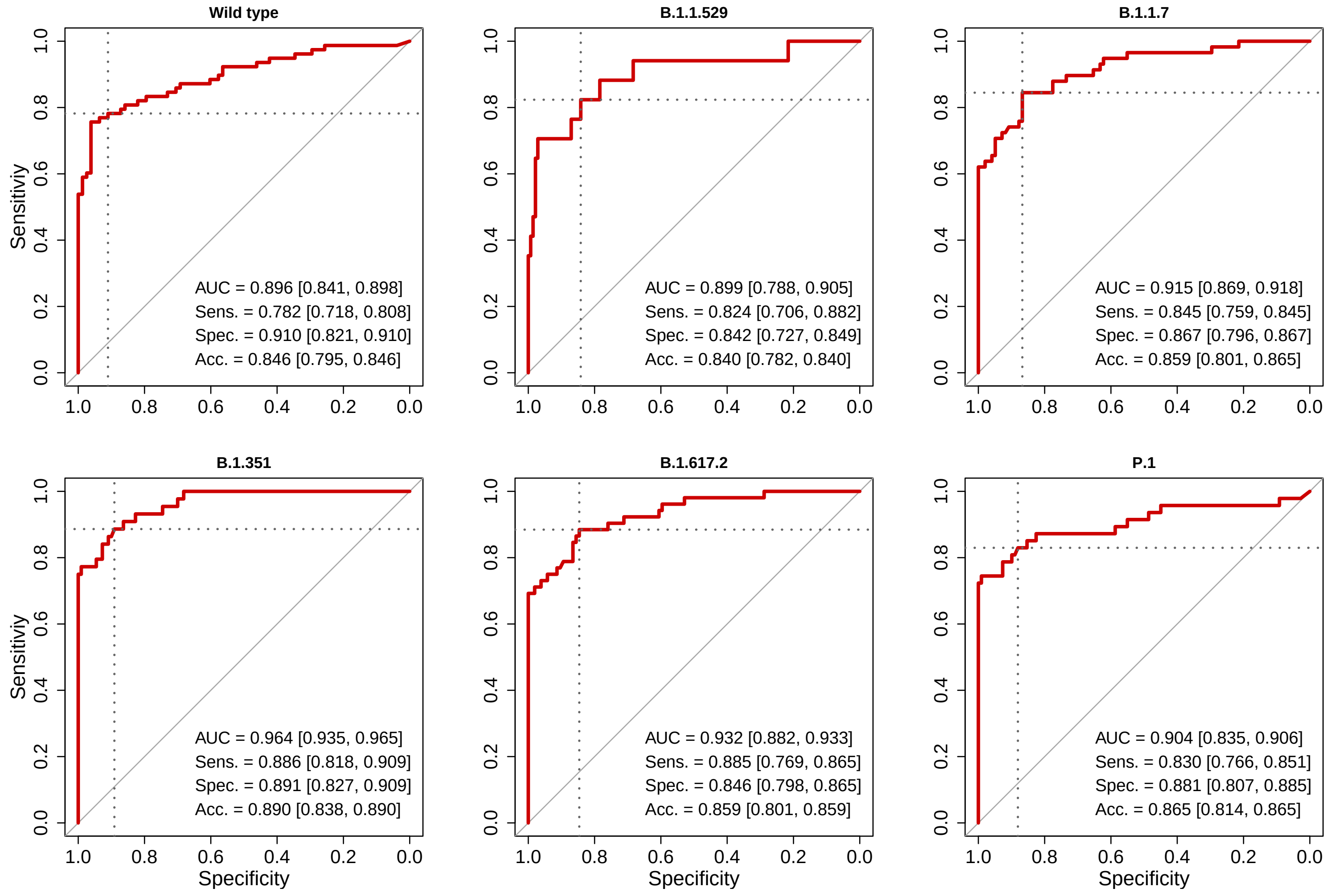

Suplementary Figure S5. Concordance of anti-S protein and neutralizing antibodies in pre-third dose samples. Receiver Operating Characteristics (ROC) and their corresponding Area Under the Curve (**AUC**) assessing the ability of anti-S protein antibody titers to predict neutralizing antibody positivity. The legends show the values of AUC, sensitivity (**Sens.**), Specificity (**Spec.)** and overall acuraccy in predicting the variant positivity as well as their corresponding bootstrap 95% confidence intervals. Total accuracy, sensitivity, and specificity are displayed for the optimal threshold, defined as the ROC point closest to the top-left part of the plot (perfect sensitivity and specificity). **AUC:** Area Under the Curve; **Sens.:** sensitivity; **Spec.:** specificity; A**cc.:** accuracy.

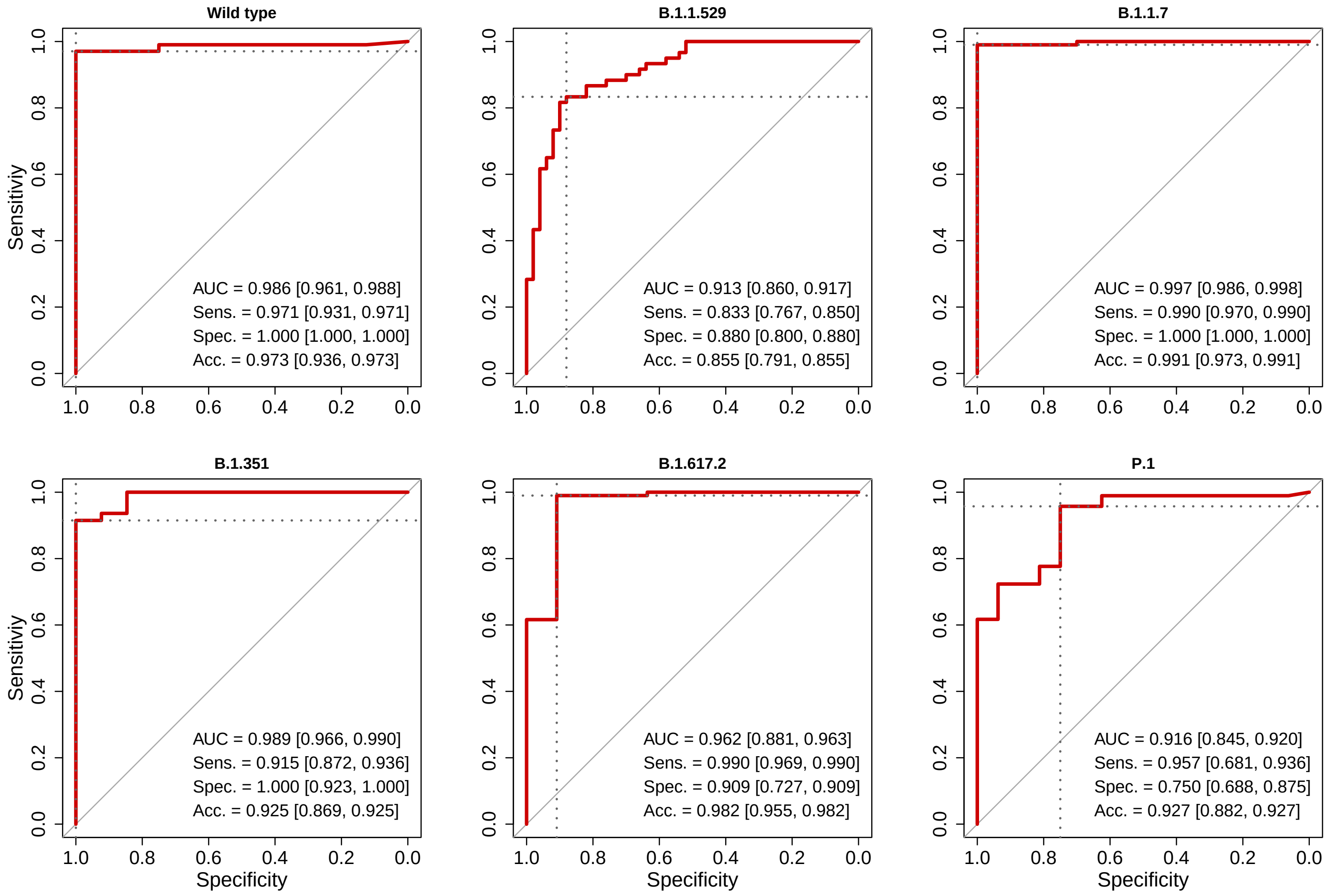

Suplementary Figure S6. Concordance of anti-S protein and neutralizing antibodies in post-third dose samples. Receiver Operating Characteristics (ROC) and their corresponding Area Under the Curve (**AUC**) assessing the ability of anti-S protein antibody titers to predict neutralizing antibody positivity. The legends show the values of AUC, sensitivity (**Sens.**), Specificity (**Spec.)** and overall acuraccy in predicting the variant positivity as well as their corresponding bootstrap 95% confidence intervals. Total accuracy, sensitivity, and specificity are displayed for the optimal threshold, defined as the ROC point closest to the top-left part of the plot (perfect sensitivity and specificity). **AUC:** Area Under the Curve; **Sens.:** sensitivity; **Spec.:** specificity; A**cc.:** accuracy.
