# Supplementary material for: Biological and glucocorticoids treatment impair the medium-term immunogenicity to SARS-CoV-2 mRNA vaccines in autoimmune inflammatory rheumatic diseases
Source: Eur J Med Res. 2024 Jan 5;29:28. doi: 10.1186/s40001-023-01620-7 (PMC10768211; doi:10.1186/s40001-023-01620-7)
Supplement: Supplementary file 2 — Additional file 2: Table S1. Patients' characteristics by sample availability for analysis. Table S2. Fold-changes (FC), boostrap 95% confidence intervals (CI) and p-values for comparison of treatment groups within each time point of sample extraction. P-values are derived from a Mann-Whitney's test. JAKi: JAK inhibitors; GCs: Glucocorticoids. Table S3. Anti-S protein antibody levels and neutralizing antibodies positivity (nABs) by rhuematic condition group before (Pre-3rd dose) and after (Post-3rd dose) of a third dose of the SARS-CoV-2 mRNA-based vaccine. Table S4. Comparison of anti-S protein antibody titers between rheumatic condition groups within each time point of sample extraction. Table S5. Increase of anti-S protein antibody levels after the third dose of the SARS-CoV-2 vaccine by treatment group. Table S6. Increase of anti-S protein antibody levels after the third dose of the SARS-CoV-2 vaccine by rheumatic condition. Table S7. Anti-S protein antibody levels by treatment group before (Pre-3rd dose) and after (Post-3rd dose) a third dose of the SARS-CoV-2 mRNA-based vaccine. Table S8 Comparison of anti-S protein antibody titers between users and non-users of Glucocroticoids (GCs) within each rheumatic condition and time point of sample extraction. Cells show Fold-changes (FC), 95% confidence intervals (CI) and p-values of the corresponding comparison. Table S9. Positivity of neutralizing antibodies by glucorcorticoids use in the overall series. Table S10. Positivity of neutralizing antibodies by glucorcorticoids use in patients under biological treatment. Table S11. Distribution of biological treatments by use of Glucocorticoids (GCs). [file 40001_2023_1620_MOESM2_ESM.pdf]

**Table S1.** Patients' characteristics by sample availability for analysis. The table presents the distribution of key parameters analyzed for all patients in our series (All, n = 157), those with pre- and post-third dose samples (Two samples available, n = 110), and those contributing to the study with an only sample collected before the third vaccine dose (One sample only, n = 47). For the later group, the table further provides descriptives for patients who failed to complete follow-up due to death (Exitus, n = 3), SARS-CoV-2 infection after the pre-third dose sample (COVID-19 infection, n = 17), and other reasons related to the study logistics (Logistics, n = 27). Cells display the medians and ranges for continuous variables (anti-S protein antibody titers and age), and the frequencies/percentages for categorical variables, including positivity for neutralizing antibodies (nABs) against diferents SARS-CoV-2 variants, such as: **Wild type, B.1.1.529** (Omicron), **B.1.1.7** (Alpha), **B.1.351** (Beta), **B.1.617.2** (Delta) and **P.1** (Gamma). The last column presents p-values from Mann-Whitney tests (continuous variables) of a Fisher's tests (categorical variables) comparing patients with and without second sample available for analyses. **SLE:** Systemic Lupus Erythematosus; **RA:** Rheumatoid Arthritis; **B27-AS:** HLA-B27 positive Ankylosing Spondylitis; **PSA:** Psoriatic Arthritis; **GCA:** Giant Cell Arteritis; **JAKi:** JAK inhibitors.

| | | **All n=157** | **Two samples available n=110 (70.1%)** | **One sample only (pre-third dose)** | | | | **P-value (Two vs One samples)** |
|---|---|---|---|---|---|---|---|---|
| | | | | **Exitus n=3 (1.9%)** | **COVID-19 n=17 (10.8%)** | **Logistics n=27 (17.2%)** | **All n=47 (29.9%)** | |
| **Anti-S-protein antibody (BAU/ml)** | | 537.0 (0.2, 87880.0) | 618.0 (0.0, 85340.0) | 233.0 (23.0, 1693.0) | 295.0 (0.2, 8672.0) | 332.0 (9.4, 87880.0) | 295.0 (0.2, 87880.0) | 0.135461 |
| **Wild type - Positive** | | 78 (50.0%) | 61 (56.0%) | 1 (33.3%) | 8 (47.1%) | 8 (29.6%) | 17 (36.2%) | 0.035666 |
| **B.1.1.529 - Positive** | | 17 (10.9%) | 15 (13.8%) | 0 (0.0%) | 0 (0.0%) | 2 (7.4%) | 2 (4.3%) | 0.097276 |
| **B.1.1.7 - Positive** | | 58 (37.2%) | 42 (38.5%) | 1 (33.3%) | 8 (47.1%) | 7 (25.9%) | 16 (34.0%) | 0.718381 |
| **B.1.351 - Positive** | | 44 (28.6%) | 34 (31.8%) | 1 (33.3%) | 4 (23.5%) | 5 (18.5%) | 10 (21.3%) | 0.245299 |
| **B.1.617.2 - Positive** | | 52 (33.3%) | 40 (36.7%) | 1 (33.3%) | 6 (35.3%) | 5 (18.5%) | 12 (25.5%) | 0.198702 |
| **P.1 - Positive** | | 47 (30.1%) | 36 (33.0%) | 2 (66.7%) | 5 (29.4%) | 4 (14.8%) | 11 (23.4%) | 0.258635 |
| **Sex - Female** | | 100 (63.7%) | 70 (63.6%) | 0 (0.0%) | 15 (88.2%) | 15 (55.6%) | 30 (63.8%) | > 0.9999 |
| **Age at third dose of SARS-CoV-2 vaccine** | | 58.8 (33.5, 88.2) | 59.2 (33.5, 88.2) | 68.7 (61.0, 83.8) | 56.8 (41.7, 85.0) | 58.2 (43.4, 79.6) | 58.3 (41.7, 85.0) | 0.557594 |
| **Rheumatic condition** | **LES** | 35 (22.3%) | 20 (18.2%) | 0 (0.0%) | 9 (52.9%) | 6 (22.2%) | 15 (31.9%) | 0.010199 |
| | **AR** | 36 (22.9%) | 32 (29.1%) | 0 (0.0%) | 0 (0.0%) | 4 (14.8%) | 4 (8.5%) | |
| | **EA B27** | 22 (14.0%) | 17 (15.5%) | 0 (0.0%) | 2 (11.8%) | 3 (11.1%) | 5 (10.6%) | |
| | **APSO** | 40 (25.5%) | 23 (20.9%) | 2 (66.7%) | 5 (29.4%) | 10 (37.0%) | 17 (36.2%) | |
| | **VGV** | 24 (15.3%) | 18 (16.4%) | 1 (33.3%) | 1 (5.9%) | 4 (14.8%) | 6 (12.8%) | |
| **Treatmen type** | **Not treated or Glucorticoids only** | 25 (15.9%) | 18 (16.4%) | 0 (0.0%) | 3 (17.6%) | 4 (14.8%) | 7 (14.9%) | 0.558644 |
| | **Immunosupressive** | 48 (30.6%) | 30 (27.3%) | 1 (33.3%) | 9 (52.9%) | 8 (29.6%) | 18 (38.3%) | |
| | **Biological** | 69 (43.9%) | 50 (45.5%) | 2 (66.7%) | 4 (23.5%) | 13 (48.1%) | 19 (40.4%) | |
| | **JAKi** | 15 (9.6%) | 12 (10.9%) | 0 (0.0%) | 1 (5.9%) | 2 (7.4%) | 3 (6.4%) | |
| **Glucocorticoids use** | | 43 (27.4%) | 31 (28.2%) | 2 (66.7%) | 5 (29.4%) | 5 (18.5%) | 12 (25.5%) | 0.845809 |
| **mRNA-based vaccine type** | **mRNA-1273** | 100 (63.7%) | 71 (64.5%) | 2 (66.7%) | 11 (64.7%) | 16 (59.3%) | 29 (61.7%) | 0.856361 |
| | **BNT162b2** | 57 (36.3%) | 39 (35.5%) | 1 (33.3%) | 6 (35.3%) | 11 (40.7%) | 18 (38.3%) | |

**Supplementary Table S2.** Fold-changes (FC), boostrap 95% confidence intervals (CI) and p-values for comparison of treatment groups within each time point of sample extraction. P-values are derived from a Mann-Whitney's test. **JAKi:** JAK inhibitors; **GCs:** Glucocorticoids.

| | **Pre-3rd dose** | | **Post-3rd dose** | |
|---|---|---|---|---|
| | **FC [95%CI]** | **P-value** | **FC [95%CI]** | **P-value** |
| **Non-biological - Not treated or GCs only** | 1.13 [0.43, 2.52] | 0.8799 | 0.95 [0.39, 3.18] | 0.9830 |
| **Biological - Not treated or GCs only** | 0.38 [0.14, 1.06] | 0.0132 | 0.70 [0.31, 2.46] | 0.5973 |
| **JAKi - Not treated or GCs only** | 0.70 [0.15, 2.14] | 0.3565 | 0.56 [0.24, 2.26] | 0.4718 |
| **Biological - Non-biological** | 0.34 [0.21, 0.72] | 0.0038 | 0.73 [0.39, 1.49] | 0.6837 |
| **JAKi - Non-biological** | 0.62 [0.18, 1.41] | 0.3329 | 0.59 [0.30, 1.47] | 0.2776 |
| **JAKi - Biological** | 1.85 [0.44, 3.85] | 0.3032 | 0.80 [0.33, 2.21] | 0.6304 |

**Supplementary Table S3.** Anti-S protein antibody levels and neutralizing antibodies positivity (nABs) by rhuematic condition group before (Pre-3rd dose) and after (Post-3rd dose) of a third dose of the SARS-CoV-2 mRNA-based vaccine. Cells show the medians and ranges of anti-S protein antibody titers, and the frequencies and percentages of positivity for nABs of diferents SARS-CoV-2 variants including: B.1.1.529 (Omicron), B.1.1.7 (Alpha), B.1.351 (Beta), B.1.617.2 (Delta) and P.1 (Gamma). The last row display the rheumatic conditions of patients represented in each treatment group and sample draw. P-values are derived from a Kruskal-Wallis tests comparing treatment groups within each sample time point. **SLE:** Systemic Lupus Erythematosus; **RA:** Rheumatoid Arthritis; **B27-AS:** HLA-B27 positive Ankylosing Spondylitis; **PSA:** Psoriatic Arthritis; **GCA:** Giant Cell Arteritis; **GCs:** Glucocorticoids; **JAKi:** JAK inhibitors.

| | **Pre-3rd dose** | | | | | | **Post-3rd dose** | | | | | |
|---|---|---|---|---|---|---|---|---|---|---|---|---|
| | **SLE n=35** | **RA n=35** | **B27-AS n=22** | **PSA n=40** | **GCA n=24** | **P-value** | **SLE n=20** | **RA n=32** | **B27-AS n=17** | **PSA n=23** | **GCA n=18** | **P-value** |
| **Anti-S-protein antibody (BAU/ml)** | 486 [0, 83890] | 335 [0, 85340] | 671 [50, 4455] | 796 [19, 9567] | 239 [0, 87880] | 0.23968 | 11323 [38, 55080] | 6433 [0, 166760] | 11712 [2342, 137780] | 14547 [888.0, 112590.0] | 4311 [0, 74400] | 0.10945 |
| **Wild type - Positive** | 21 (60.0%) | 21 (60%%) | 19 (86.4%) | 30 (75.0%) | 12 (50%) | 0.04812 | 19 (95.0%) | 30 (93.8%) | 17 (100%) | 23 (100%) | 16 (88.9%) | 0.25326 |
| **B.1.1.529 - Positive** | 6 (17.1%) | 3 (8.6%) | 1 (4.5%) | 3 (7.5%) | 4 (16.7%) | 0.47496 | 11 (55.0%) | 18 (56.2%) | 9 (52.9%) | 16 (69.6%) | 6 (33.3%) | 0.25326 |
| **B.1.1.7 - Positive** | 18 (51.4%) | 11 (31.4%) | 8 (36.4%) | 15 (37.5%) | 6 (25.0%) | 0.29535 | 18 (90.0%) | 28 (87.5%) | 17 (100.0%) | 22 (95.7%) | 15 (83.3%) | 0.43294 |
| **B.1.351 - Positive** | 15 (42.9%) | 8 (23.5%) | 6 (27.3%) | 10 (25.0%) | 5 (21.7%) | 0.35435 | 17 (89.5%) | 27 (87.1%) | 15 (93.8%) | 21 (91.3%) | 14 (77.8%) | 0.69225 |
| **B.1.617.2 - Positive** | 17 (48.6%) | 11 (31.4%) | 7 (31.8%) | 11 (27.5%) | 6 (25.0%) | 0.30516 | 18 (90.0%) | 28 (87.5%) | 17 (100.0%) | 21 (91.3%) | 15 (83.3%) | 0.57836 |
| **P.1 - Positive** | 15 (42.9%) | 12 (34.3%) | 4 (18.2%) | 10 (25.0%) | 6 (25.0%) | 0.28335 | 17 (85.0%) | 27 (84.4%) | 15 (88.2%) | 20 (87.0%) | 15 (83.3%) | 0.9999 |

**Supplementary Table S4.** Comparison of anti-S protein antibody titers between rheumatic condition groups within each time point of sample extraction. Cells show Fold-changes (FC), 95% confidence intervals (CI) and p-values of the corresponding comparison. FCs and p-values were derived from a mixed-effects linear model where sex, age, rheumatic condition, treatment type, use of glucocorticoids, type of vaccine, sample type (pre- or post-3rd dose) and time from previous dose where included as fixed effects, as well as the interaction between: sex and sample type; treatment and sample type; rheumatic condition and sample type; use of glucocorticoids and sample type; time from 2nd and third vaccine dose and sample type; glucocorticoids use and treatment regimen; and glucorticoids use and rheumatic condition. Individual effects were included as random effects in this model. **SLE:** Systemic Lupus Erythematosus; **RA:** Rheumatoid Arthritis; **B27-AS:** HLA-B27 positive Ankylosing Spondylitis; **PSA:** Psoriatic Arthritis; **GCA:** Giant Cell Arteritis; **JAKi:** JAK inhibitors; **GCs:** Glucocorticoids; **FC:** Fold-Change; **95%CI:** 95% confidence interval.

| | **Pre-3rd dose** | | **Post-3rd dose** | |
|---|---|---|---|---|
| | **FC [95%CI]** | **P-value** | **FC [95%CI]** | **P-value** |
| **RA - SLE** | 3.31 [0.87, 12.60] | 0.0788 | 2.58 [0.64, 10.43] | 0.1844 |
| **B27-AS - SLE** | 3.21 [0.85, 12.13] | 0.0850 | 1.34 [0.33, 5.51] | 0.6860 |
| **PSA - SLE** | 8.27 [2.08, 32.80] | 0.0027 | 3.74 [0.85, 16.59] | 0.0821 |
| **GCA - SLE** | 5.14 [1.15, 22.94] | 0.0317 | 1.31 [0.27, 6.50] | 0.7373 |
| **B27-AS - RA** | 0.97 [0.20, 4.60] | 0.9695 | 0.52 [0.10, 2.81] | 0.4469 |
| **PSA - RA** | 2.50 [0.79, 7.93] | 0.1208 | 1.45 [0.38, 5.52] | 0.5830 |
| **GCA - RA** | 1.55 [0.38, 6.36] | 0.5406 | 0.51 [0.11, 2.39] | 0.3933 |
| **PSA - B27-AS** | 2.57 [0.57, 11.60] | 0.2186 | 2.80 [0.53, 14.64] | 0.22353 |
| **GCA - B27-AS** | 1.60 [0.31, 8.28] | 0.57438 | 0.98 [0.16, 6.05] | 0.9840 |
| **GCA - PSA** | 0.62 [0.15, 2.53] | 0.5074 | 0.35 [0.07, 1.77] | 0.2054 |

**Supplementary Table S5.** Increase of anti-S protein antibody levels after the third dose of the SARS-CoV-2 vaccine by treatment group. Cells show the Fold-changes (FC), bootstrap 95% confidence intervals (CI) and p-values comparing the the patients' titer levels after and before the administration of the third vaccine's dose. P-values are derived from a Wilcoxon test using only patients with both samples available. **JAKi:** JAK inhibitors; **GCs:** Glucocorticoids.

| | **FC [95%CI]** | **P-value** |
|---|---|---|
| **Not treated or GCs only** | 16.89 [3.97, 50.67] | 0.0066 |
| **Non-biological** | 11.26 [5.63, 19.19] | < 0.0001 |
| **Biological** | 22.35 [8.78, 49.27] | < 0.0001 |
| **JAKi** | 10.51 [3.22, 32.23] | 0.02686 |

**Supplementary Table S6.** Increase of anti-S protein antibody levels after the third dose of the SARS-CoV-2 vaccine by rheumatic condition. Cells show the Fold-changes (FC), bootstrap 95% confidence intervals (CI) and p-values comparing the the patients' titer levels after and before the administration of the third vaccine's dose. P-values are derived from a Wilcoxon test using only patients with both samples available.

| | FC [95%CI] | P-value |
|---|---|---|
| **Systemic Lupus Erythematosus** | 23.30 [7.21, 62.25] | 0.0010 |
| **Rheumatoid Arthritis** | 19.20 [6.71, 38.39] | < 0.0001 |
| **HLA-B27 positive Ankylosing Spondylitis** | 17.45 [6.99, 35.33] | < 0.0001 |
| **Psoriatic Arthritis** | 18.28 [7.89, 41.83] | < 0.0001 |
| **Giant Cell Arteritis** | 18.07 [6.28, 80.06] | 0.0373 |

**Supplementary Table S7.** Anti-S protein antibody levels by treatment group before (Pre-3rd dose) and after (Post-3rd dose) a third dose of the SARS-CoV-2 mRNA-based vaccine. Cells show the medians an ranges of anti-S protein antibody titers. P-values are derived from a Mann-Whiteny test comparing treatment groups within each sample time point. **JAKi:** JAK inhibitors; **GCs:** Glucocorticoids.

| | **Pre-3rd dose** | | | **Post-3rd dose** | | |
|---|---|---|---|---|---|---|
| | **No GCs** | **Gcs** | **P-value** | **No GCs** | **Gcs** | **P-value** |
| **Not treated or GCs only** | 1522 [152, 83890] | 300 [43, 20363] | 0.0272 | 11712 [2037, 55080] | 3881 [1002, 74400] | 0.6272 |
| **Non-biological** | 996 [14, 9567] | 218 [16, 40700] | 0.0538 | 10338 [2246, 112590] | 11643 [433, 33200] | 0.8512 |
| **Biological** | 419 [0, 12069] | 72 [0, 87880] | 0.0402 | 10890 [0, 137780] | 1447 [0, 23022] | 0.0124 |
| **JAKi** | 882 [34, 85340] | 274 [43, 967] | 0.2030 | 10229 [1479, 166760] | 4378 [393, 16823] | 0.1495 |

**Supplementary Table S8** Comparison of anti-S protein antibody titers between users and non-users of Glucocroticoids (GCs) within each rheumatic condition and time point of sample extraction. Cells show Fold-changes (FC), 95% confidence intervals (CI) and p-values of the corresponding comparison. FCs and p-values were derived from a mixed-effects linear model where sex, age, rheumatic condition, treatment type, use of glucocorticoids, type of vaccine, sample type (pre- or post-3rd dose) and time from previous dose where included as fixed effects, as well as the interaction between: sex and sample type; treatment and sample type; rheumatic condition and sample type; use of glucocorticoids and sample type; time from 2nd and third vaccine dose and sample type; glucocorticoids use and treatment regimen; and glucorticoids use and rheumatic condition. Individual effects were included as random effects in this model. **SLE:** Systemic Lupus Erythematosus; **RA:** Rheumatoid Arthritis; **B27-AS:** HLA-B27 positive Ankylosing Spondylitis; **PSA:** Psoriatic Arthritis; **GCA:** Giant Cell Arteritis; **JAKi:** JAK inhibitors; **GCs:** Glucocorticoids; **FC:** Fold-Change; **95%CI:** 95% confidence interval.

| | **Pre-3rd dose** | | **Post-3rd dose** | |
|---|---|---|---|---|
| | **FC [95%CI]** | **P-value** | **FC [95%CI]** | **P-value** |
| **SLE** | 0.25 [0.05, 1.25] | 0.0911 | 0.39 [0.07, 2.20] | 0.2832 |
| **RA** | 0.46 [0.11, 1.89] | 0.2837 | 0.71 [0.17, 2.91] | 0.6384 |
| **PSA** | 0.85 [0.17, 4.24] | 0.8435 | 1.31 [0.23, 7.47] | 0.7639 |
| **CGA** | 0.25 [0.04, 1.69] | 0.1563 | 0.39 [0.05, 2.82] | 0.3494 |

**Supplementary Table S9.** Positivity of neutralizing antibodies by glucorcorticoids use in the overall series. Cells show, for different variants of the SARS-CoV-2, the absolute frequency and the percentage of positive immune response, and the p-value for the comparison of this frequencies derived from a exact Fisher's test. Variants of SARS-Cov-2 are: **B.1.1.529** (Omicron), **B.1.1.7** (Alpha), **B.1.351** (Beta), **B.1.617.2** (Delta) and **P.1** (Gamma). **GCs:** Glucocorticoids.

| | **Pre-3rd dose** | | | **Post-3rd dose** | | |
|---|---|---|---|---|---|---|
| | **No GCs N(%)** | **GCs N(%)** | **P-value** | **No GCs N(%)** | **GCs N(%)** | **P-value** |
| **Wild type** | 84 (73.7%) | 19 (45.2%) | 0.0012 | 77 (97.5%) | 28 (90.3%) | 0.1349 |
| **B.1.1.529** | 12 (10.5%) | 5 (11.9%) | 0.7780 | 47 (59.5%) | 13 (41.9%) | 0.1359 |
| **B.1.1.7** | 47 (41.2%) | 11 (26.2%) | 0.0955 | 76 (96.2%) | 24 (77.4%) | 0.0050 |
| **B.1.351** | 37 (32.7%) | 7 (17.1%) | 0.0698 | 71 (93.4%) | 23 (74.2%) | 0.0097 |
| **B.1.617.2** | 43 (37.7%) | 9 (21.4%) | 0.0588 | 75 (94.9%) | 24 (77.4%) | 0.0109 |
| **P.1** | 38 (33.3%) | 9 (21.4%) | 0.1726 | 71 (89.9%) | 23 (74.2%) | 0.0670 |

**Supplementary Table S10.** Positivity of neutralizing antibodies by glucorcorticoids use in patients under biological treatment. Cells show, for different variants of the SARS-CoV-2, the absolute frequency and the percentage of positive immune response, and the p-value for the comparison of this frequencies derived from a exact Fisher's test. Variants of SARS-Cov-2 are: **B.1.1.529** (Omicron), **B.1.1.7** (Alpha), **B.1.351** (Beta), **B.1.617.2** (Delta) and **P.1** (Gamma). **GCs:** Glucocorticoids.

| | **Pre-3rd dose** | | | **Post-3rd dose** | | |
|---|---|---|---|---|---|---|
| | **No GCs N(%)** | **GCs N(%)** | **P-value** | **No GCs N(%)** | **GCs N(%)** | **P-value** |
| **Wild type** | 34 (60.7%) | 3 (25.0%) | 0.0299 | 40 (95.2%) | 5 (62.5%) | 0.0242 |
| **B.1.1.529** | 2 (3.6%) | 1 (8.3%) | 0.4469 | 24 (57.1%) | 1 (12.5%) | 0.0488 |
| **B.1.1.7** | 17 (30.4%) | 2 (16.7%) | 0.4866 | 40 (95.2%) | 4 (50.0%) | 0.0039 |
| **B.1.351** | 10 (18.2%) | 2 (16.7%) | 0.9999 | 37 (90.2%) | 4 (50.0%) | 0.0171 |
| **B.1.617.2** | 14 (25.0%) | 2 (16.7%) | 0.7171 | 39 (92.9%) | 4 (50.0%) | 0.0085 |
| **P.1** | 12 (21.4%) | 3 (25.0%) | 0.7188 | 39 (92.9%) | 3 (37.5%) | 0.0012 |

**Table S11.** Distribution of biological treatments by use of Glucocorticoids (GCs).

| | No GCs | GCs |
|---|---|---|
| **anti-TFN** | 21 (87.5%) | 3 (12.5%) |
| **Baricitinib** | 0 (0.0%) | 1 (100.0%) |
| **Belimumab** | 3 (75.0%) | 1 (25.0%) |
| **IL-17** | 17 (94.4%) | 1 (5.6%) |
| **Mycophenolate mofetil** | 2 (100.0%) | 0 (0.0%) |
| **Rituximab** | 4 (100.0%) | 0 (0.0%) |
| **Sulfasalazine** | 0 (0.0%) | 1 (100.0%) |
| **Tocilizumab** | 10 (62.5%) | 6 (37.5%) |
| **Ustekinumab** | 1 (50.0%) | 1 (50.0%) |
